# Multi-class/residue method for determination of veterinary drug residues, mycotoxins and pesticide in urine using LC-MS/MS technique

**DOI:** 10.1186/s12917-023-03720-2

**Published:** 2023-09-14

**Authors:** Zehra Hajrulai-Musliu, Risto Uzunov, Stefan Jovanov, Dea Musliu, Elizabeta Dimitrieska-Stojkovikj, Biljana Stojanovska-Dimzoska, Aleksandra Angeleska, Velimir Stojkovski, James Jacob Sasanya

**Affiliations:** 1 Faculty of Veterinary Medicine-Skopje, “Ss. Cyril, Methodius” University in Skopje, Lazar Pop-Trajkov 5/7, Skopje, 1000 Republic of North Macedonia; 2 Faculty of Pharmacy, “Ss. Cyril and Methodius” University in Skopje, Majka Tereza 47, Skopje, 1000 Republic of North Macedonia; 3 International Atomic Energy Agency, Vienna International Centre, P. O. Box 100, Vienna, A-1400 Austria

**Keywords:** Veterinary drugs, Mycotoxins, Pesticides, Urine, LC-MS/MS, Method development

## Abstract

**Background:**

Veterinary drugs are widely used in animals to prevent diseases and are a complex set of drugs with very different chemical properties. Multiclass and multi-residue methods for simultaneous detection of residues from veterinary drugs and contaminants in urine are very rare or non-existent. Therefore, the aim of this study was to develop and validate a sensitive and reliable quantitative LC-MS/MS method for simultaneous determination of a wide range of veterinary drug and pesticide residues and mycotoxins in bovine urine. This involved 42 veterinary drug residues (4 thyreostats, 6 anabolic hormones, 2 lactones, 10 beta agonists, 15 antibiotics, 5 sulphonamides), 28 pesticides and 2 mycotoxins. Stable isotopically labelled internal standards were used to facilitate effective quantification of the analytes. Analysis was performed in both positive and negative ionization modes with multiple reaction monitoring transitions over a period of 12 min.

**Results:**

The parameters validated included linearity, limit of detection (LOD), limit of quantification (LOQ), detection capability (CCβ), decision limit (CCα), stability, accuracy and precision. The process followed guidelines of the regulation 2021/808/EC. The calibration curves were linear with coefficient of correlation (R^2^) from 0.991 to 0.999. The LODs were from 0.01 to 2.71 µg/L, while the LOQs were from 0.05 to 7.52 µg/L. The CCα and CCβ were in range 0.05–12.11 µg/L and 0.08–15.16 µg/L. In addition, the average recoveries of the spiked urine samples were from 71.0 to 117.0% and coefficient of variation (CV) < 21.38% (intraday and interday).

**Conclusion:**

A new isotopic LC-MS/MS method has been developed, validated and applied for identification and quantification of 72 residues of veterinary drugs and pesticides and other contaminants such as mycotoxins in bovine urine. The most appropriated sample preparation procedures involved sodium acetate buffer, enzymatic hydrolysis using β-glucuronidase and cleanup solid phase extraction with OASIS SPE cartridges. The parameters were satisfactorily validated fulfilling requirements under Regulation 2021/808/EC. Consequently, the method could be used in routine analysis of bovine urine samples for simultaneous detection of veterinary drug and pesticide residues as well as contaminants such as mycotoxins.

## Introduction

Veterinary drugs are widely used in livestock production to prevent or treat diseases. While in some countries certain veterinary drugs such as thyreostats, anabolic hormones and β-agonists may be used as a growth-promoting agents [1]. This is generally prohibited in other regions such as the European Union. The residues of veterinary drugs in animal tissues/matrices resulting from incorrect use and/or nonobservance of withdrawal period can affect humans [2]. To ensure food safety and safeguard human health, the monitoring of residues in animal products and live animals is very important [3–4]. The measures for monitoring of these residues in live animals and animal products are prescribed in Regulation (EU) 2017/625 [5]. An advantage of analyzing urine or blood from live animals for presence of veterinary drug residues is the possibility to retest animals in case of a suspect result. Veterinary drugs are metabolized by animals, and some of the drugs remain in the animal body, while others enter the environment through excreta. Also, generally the drug concentration in urine tends to be higher than in the muscle or other tissues [6].

Animals are also often simultaneously exposed to other hazards such as mycotoxins pesticides or heavy metals, and since these may also end up in food consumption, multi-residue and multi-toxin exposure studies are therefore very relevant to public health. Appropriate analytical methods are therefore required [7]. The determination of the multiple substances in biological matrices such as urine and blood from live animals could be reliable and useful in exposure assessment (short- and long-term) and may predict future effects on human health [8].

The establishment of sensitive analytical methods to detect residues and contaminants in food from animal origin as well as live animals is important for safety and public health. Currently, more sensitive methods for the determination of residues and contaminants in food of animal origin have been developed, but there are few multi-residue and multiclass analytical methods for simultaneous detection of residues of veterinary drugs and other hazards such us pesticides and mycotoxins. Zhan et al., (2013) [9], developed an LC–MS/MS method for screening for multi-class veterinary drug residues and other contaminants in muscle while Hajrulai-Musliu et al., (2021), reported an LC–MS/MS method for multiple residues and contaminants in bovine meat [10]. Danezis et al., (2016) [11], developed a HILIC chromatography-MS/MS method for detection of pesticides, plant hormones, veterinary drugs and mycotoxins in various food matrices while Gómez-Pérez et al., (2015) [12], published a method for analysis of pesticide and veterinary drug residues in baby food by liquid chromatography coupled to Orbitrap high resolution mass spectrometry, as well as Wei et al., (2015) [13], who developed multi-residue screening method for analysis of veterinary drugs, their metabolites and pesticides in meat by LC-MS/MS. Xie et al., (2015), developed LC-MS/MS methods for analysis of veterinary drugs, pesticides and mycotoxins in dairy products [14].

Multiclass and multi-residue methods for simultaneous detection of residues from veterinary drugs and contaminants in urine are very rare or non-existent. A range of methods for residues of veterinary drugs and contaminants have been published from different authors although these involve single class or groups [15–22]. Stanley and Foo (2006) published a multiresidue method for simultaneous screening of more than 250 veterinary drugs alone in equine urine [23]. The multiclass/residue methods present several advantages to testing laboratories and monitoring programs [10, 24].

The objective of this study was to develop and validate a sensitive and reliable quantitative LC-MS/MS method for simultaneous determination of a wide range of veterinary drug and pesticide residues and mycotoxins in bovine urine. This involved 72 analyses including 42 veterinary drug residues (4 thyreostats, 6 anabolic hormones, 2 lactones, 10 beta agonists, 15 antibiotics, 5 sulphonamides), 28 pesticides and 2 mycotoxins. Analysis was performed in both positive and negative ionization modes with multiple reaction monitoring transitions over a period of 12 min.

Six extraction protocols for effective and rugged multiresidue extraction of 72 compounds from urine were investigated. Four of the protocols followed solid phase extraction (two with and two without enzymatic hydrolysis), while the other two protocol involved usage of liquid-liquid extraction (also with and without enzymatic hydrolysis). The parameters validated included: limit of detection (LOD), limit of quantification (LOQ), decision limit (CCα), detection capability (CCβ), linearity, accuracy and precision. The criteria prescribed in Regulation 2021/808/EC were followed [25].

## Materials and methods

### Chemicals and reagents

Methanol, acetonitrile and water (LC-MS/MS grade), ethyl acetate, dichloromethane, ammonium hydroxide, acetic acid, ammonium acetate (HPLC grade) were purchased from Carlo Erba Reagent S.A.S (Val de Reuil, France); formic acid (LC-MS/MS grade), sodium acetate (p.a.), sodium dihydrogen phosphate hydrate (p.a.), disodium hydrogen phosphate dihydrate (p.a.), sodium chloride (p.a.), β-glucuronidase aryl sulfatase from Helix pomatia and trichloroacetic acid (≥ 99.5%), Discovery® DSC-MCAX cartridges (300 mg/6 ml) were from Merck (Darmstadt, Germany) and Oasis HLB cartridges (500 mg/6ml) were from Waters (Milford, MA, USA).

### Standards and isotopically-labelled internal standards

#### The analytical standards and purity levels included

Thyreostats: thiouracil (100%), propylthiouracil (99.6%), methylthiouracil (≥ 98.0%) and tapazol (99.7%) were purchased from Sigma-Aldrich (St. Louis, MO, USA); Anabolic steroids: 19 nortestosterone (99.8%), clostebol (99.1%), boldenone (99.1%), methyltestosterone (99.5%) and testosterone (100.0%) were purchased from Sigma-Aldrich (St. Louis, MO, USA), while stanozolol (99.8%) were obtained from Dr. Ehrenstorfer GmbH (Augsburg, Germany); Lactones of resorcylic acid: taleranol (99.5%) was purchased from Sigma-Aldrich (St. Louis, MO, USA), while zeranol (99.9%) was obtained from Dr. Ehrenstorfer GmbH (Augsburg, Germany); β-agonists: clenbuterol HCl (99.1%), isoxsuprine HCl (100%), salbutamol (99.4%), zilpaterol HCl (96.0%), ractopamine HCl (95.5%) and terbutaline hemisulfate salt (100.0%) were purchased from Sigma-Aldrich (St. Louis, MO, USA), while brombuterol (98.0%), mabuterol HCl (98.0%), cimbuterol (98.0%) and clenpenterol HCl (98.0%) were obtained from Witega (Berlin, Germany); Antimicrobials: Amoxicillin (99.6%), ampicillin (99.8%), benzylpenicillin (99.3%), cloxacillin (98.7%), oxacillin (98.4%), lincomycin (100.3%), tylosin (87.9%), trimethoprim (99.5%), tetracycline (96.8%) and cephapirin (98.5%) were purchased from Sigma-Aldrich (St. Louis, MO, USA) and the rest of antimicrobials: ceftiofur (98.01%), cephalexin (96.6%), oxytetracycline (96.5%), enrofloxacin (99.74%), ciprofloxacin (98.0%), sulfadimidine (99.6%), sulfamethoxazole (99.7%), sulfadiazine (99.8%), sulfachloropyridazine (99.1%) and sulfadimethoxine (99.7%) were obtained from Dr. Ehrenstorfer GmbH (Augsburg, Germany); Pesticides: carbofuran (99.9%), carbaryl (99.9%), parathion (99.7%), malathion (99.2%), diazinon (98.3%), dimethoate (99.8%), atrazine (99.5%), cypermethrin (98.4%), permethrin (98.1%), deltamethrin (99.9%), coumaphos (99.7%), dicholphos (99.8%), chlorpyrifos (99.8%), boscalid (99.5%), fentoate (98.8%), fenthion (98.5%), fenvalerate (99.4%), monocrotophos (99.8%), malaoxon (99.0%), methamidophos (98.1%), metacrifos (96.1%), amitraz (99.8%), omethoate (98.4%), vamidothion (≥ 98.0%), phosmet (99.8%), heptenophos (98.7%), bifenthrin (99.0%), methomyl (99.0%) were purchased from Sigma-Aldrich (St. Louis, MO, USA); Mycotoxins: Ochratoxin A (≥ 98.0%) and zearalenone (99.0%) were obtained from Trilogy Analytical Laboratory, Inc. (Washington, USA).

The isotopic labelled internal standards used were: anabolic steroid: 19 − 17 β Nortestosteron-d3 was obtained from the RIVM, Netherlands; lactones of resocylic acid: β-zearalenol-d4 (≥ 98.0%) was obtained from Toronto Research Chemicals Inc. (Toronto, Canada); β agonists: Clenbuterol-d6 HCl (98.0%), brombuterol-d9 HCl (98.0%), mabuterol-d9 HCl (98.0%), clenpenterol-d5 HCl (98.0%) and cimbuterol-d9 (98.0%) obtained from Witega (Berlin, Germany), isoxsuprine-d5 hemifumarate (≥ 98.0%) and ractopamine-d6 HCl (≥ 98.0%) from the European reference laboratory (EURL) at RIKILT, The Netherlands, salbutamol (albuterol)-d9 (≥ 98.0%) from Dr. Ehrenstorfer GmbH (Augsburg, Germany), zilpaterol–d7 (≥ 98.0%) was obtained from Toronto Research Chemicals Inc. (Toronto, Canada), while terbutaline-d9 acetate hemihydrate (99.3%) was obtained from Sigma-Aldrich (St. Louis, MO, USA); antimicrobials: flunixin–d3 (100.0%), penicillin G-d7 N-ethylpiperidinium (98.1%) salt and pesticides: atrazine-d5 (99.7%), chlorpyrifos-d10 (100%) and carbofuran-d3 (99.3%) were obtained from Sigma-Aldrich (St. Louis, MO, USA) [10, 26].

### Preparation of stock standard solutions

To prepare the stock standards solutions, 5 to 10 mg of each standard and internal standard was weighed and transferred to a 10 ml volumetric flask which contained methanol. The concentration of individual stock solutions was in range from 0.5 mg/ml to 1.0 mg/ml. The solutions were stored at -20 °C.

### Preparation of working standard solutions

A total of five mixed working solutions were prepared in methanol from the standards stock solutions. According to the values for minimum method performance requirements (MMPRs) the standards and internal standards were divided in groups [27]. The standards from analytes with the same values for MMPRs (Table 1) were placed in the same group, while standards from analytes without MMPSs and MRL were placed in another group, because they had similar sensitivity levels. Group 1 (mixed working solution 1) consisted of: thiouracil, methylthiouracil, propylthiouracil and tapazole; group 2 (mixed working solution 2) had: methyltestosterone, 19 nortestosterone, stanozolol, isoxsuprine, ractopamine, salbutamol, zilpaterol, terbutaline; group 3 (mixed working solution 3) consisted of: clenbuterol, brombuterol, mabuterol, clenpenterol and cimbuterol; group 4 (mixed working solution 4) contained: boldenone, zeranol and taleranol and the rest of the analyses were placed in group 5 (mixed working solution 5). The initial concentration of all five mixed working solutions which were prepared from individual standard stock solutions was 10 µg/ml, but this was diluted further while preparing the calibration curve, depending on the required concentrations. From the appropriate mixed working solutions from 10 µg/ml were prepared the next mixed working solutions: for group 1: 1.0 µg/ml (prepared from mixed working solution 1), for group 2: 1.0 µg/ml and 0.1 µg/ml (prepared from mixed working solution 2), for group 3: 1.0 µg/ml and 0.01 µg/ml (prepared from mixed working solution 3), for group 4: 1.0 µg/ml and 0.1 µg/ml (prepared from mixed working solution 4) and for group 5: 1.0 µg/ml ((prepared from mixed working solution 5); (the solutions were prepared in the volumetric flask from 10 ml and dissolved in methanol)). The mixed working solutions were used for preparation of matrix match calibration curve and spiking of the urine samples. Matrix match calibration curves were prepared in blank bovine urine samples (Table 2).

**Table 1:** MMPR values for target analytes included in the study

Analytes	MMPR (µg/L)
Thiouracil (TU)	10
Methylthiouracil (MTU)	10
Propylthiouracil (PTU)	10
Tapazole (TAP)	10
Testosterone (TEST)	/
Methyltestosterone (MEST)	0.5
Boldenone (BOLD)	1
19 Nortestosterone (19 NO)	0.5
Stanozolol (STZL)	0.5
Clostebol (CLBL)	/
Zeranol (ZENL)	1
Taleranol (TANL)	1
Clenbuterol (CLEN)	0.1
Brombuterol (BROM)	0.1
Mabuterol (MABT)	0.1
Clenpenterol (CLEP)	0.1
Isoxuprin (ISOX)	0.5
Cimbuterol (CIMB)	0.1
Ractopamine (RACT0	0.5
Salbutamol (SALB)	0.5
Zilpaterol (ZILP)	0.5
Terbutaline (TERB)	0.5
Amoxicillin (AMOX)	/
Ampicillin (AMP0	/
Benzylpenicillin (BNPC)	/
Lincomycin (LINK)	/
Tylosin (TYLS)	/
Trimethoprim (TRIP)	/
Cephapirin (CEPR)	/
Tetracycline (TETC)	/
Cloxacillin (CLCN)	/
Oxacillin (OXIN)	/
Cefalexin (CEFA)	/
Ceftiofur (CEFT)	/
Enrofloxacin (ENRO)	/
Ciprofloxacin (CIPR)	/
Oxytetracycline (OXTT)	/
Sulfachloropyridazine (SUPZ)	/
Sulfadiazine (SUDI)	/
Sulfadimethoxine (SUDM)	/
Sulfadimidine (SULD)	/
Sulfamethoxazole (SULM)	/
Carbofuran (CRL)	/
Carbaryl (CRB)	/
Parathion (PTN)	/
Malathion (MTN)	/
Diazinon (DNN)	/
Dimethoate (DIM)	/
Atrazine (ATRZ)	/
Permethrine (PEMT)	/
Cypermethrine (CIRM)	/
Deltamethrine (DELM)	/
Coumaphos (COU)	/
Dichlorophos (DIRP)	/
Chlorpyrifos (CHRS)	/
Fenvalerate (FERT)	/
Boscalid (BOS)	/
Fenthoate (FETE)	/
Fenthione (FEON)	/
Monocrotophos (MOCR)	/
Malaoxon (MAON)	/
Methamidophos MEDF	/
Metacrifos (MECF)	/
Amitraz (AMRZ)	/
Omethoate (OMAT)	/
Vamidothion (VAON)	/
Phosmet (FOST)	/
Heptenophos (HEPH)	/
Bifenthrin (BFNT)	/
Methomyl (MEML)	/
Zearalenone (ZEAN)	/
Ochratoxin A (OTAA)	/

The mixed working solutions from internal standards were prepared in methanol from the internal standards stock solutions. The internal standards were also divided in two groups, the first one (with concentration of 5 µg/l in the samples, after spiking) consisted of: 19 − 17β Nortestosteron-d3, isoxsuprine-d5, ractopamine-d6, salbutamol-d9, zilpaterol-d7, terbutaline-d9, Clenbuterol-d6, brombuterol-d9, mabuterol-d9, clenpenterol-d5, cimbuterol-d9 and β-zearalenol-d4. The concentration of the mixed internal standard working solution was 10 µg/ml, from which additionally, for spiking of the samples and preparation of matrix match calibration curve, was prepared mixed internal standard working solution with concentration from 1000 ng/ml. The second group (with concentration of 50 µg/l in the samples, after spiking) consisted of: flunixin–d3, penicillin G-d7, atrazine-d5, chlorpyrifos-d10 and carbofuran-d3. The concentration of this mixed internal standard working solution for spiking of the samples and preparation of matrix match calibration curve was 10 µg/ml.

Matrix-matched calibration prepared in blank bovine urine and internal standard were utilized for quantification to compensate the matrix effects that influence analytical response. Due to aspects of availability, cost, and convenience the use of internal standards was feasible only for β-agonists, 7 antibiotics, 3 pesticides, 1 anabolic steroid and 1 mycotoxin (Table 3).

**Table 2:** Sample preparation steps

Sample preparation steps	Initial sample preparation	Spiking	Addition of buffer	Enzymatic hydrolyze	Centrifugation	LLE	SPE	Evaporation	Reconstitution	LC-MS/MS analysis
Protocol no										
1	**+**	**+**	**+**	**-**	**+**	**+**	**-**	**+**	**+**	**+**
2	**+**	**+**	**+**	**+**	**+**	**+**	**-**	**+**	**+**	**+**
3	**+**	**+**	**+**	**-**	**+**	**-**	+*	**+**	**+**	**+**
4	**+**	**+**	**+**	**+**	**+**	**-**	+*	**+**	**+**	**+**
5	**+**	**+**	**+**	**-**	**+**	**-**	+**	**+**	**+**	**+**
6	**+**	**+**	**+**	**+**	**+**	**-**	+**	**+**	**+**	**+**

*SPE with OASIS HLB cartridges; **SPE with DSC-MCAX cartridges

**Table 3:** MRM parameters for the target analytes

Standard	Polarity	Precursor ion (m/z)	Product ion (m/z)	Collision energy	Cone voltage	Retention time
Thiouracil (TU)	+	128.80	112.0 69.86	20 18	30	4.90
Methylthiouracil (MTU)	+	142.83	125.90 83.85	18 22	30	2.35
Propylthiouracil (PTU)	+	170.88	154.30 111.91	20 24	32	3.91
Tapazole (TAP)	+	114.82	110.15 87.83	16 16	36	1.23
Testosterone (TEST)	+	289.16	108.99 96.95	24 28	36	7.70
Methyltestosterone (MEST)	+	303.22	96.96 109.0	28 24	36	7.79
Boldenone (BOLD)	+	287.16	121.03 135.02	24 16	34	7.50
19 Nortestosterone (19 NO)	+	275.14	80.56 109.0	34 26	38	7.32
Stanozolol (STZL)	+	329.22	80.95 95.00	46 46	64	7.90
Clostebol (CLBL)	+	323.16	130.98 142.96	26 26	40	7.98
Zeranol (ZENL)	-	321.03	90.87 40.90	40 40	74	6.83
Taleranol (TANL)	-	321.03	90.87 40.90	34 40	74	7.48
Clenbuterol (CLEN)	+	276.97	202.95 131.87	16 30	22	4.89
Brombuterol (BROM)	+	366.90	292.84 211.42	20 34	26	5.41
Mabuterol (MABT)	+	310.95	236.99 216.96	18 26	24	5.26
Clenpenterol (CLEP)	+	291.00	202.92 131.89	16 30	28	5.62
Isoxuprin (ISOX)	+	302.04	106.96 164.01	30 16	26	6.29
Cimbuterol (CIMB)	+	234.03	159.98 142.94	16 28	22	3.01
Ractopamine (RACT)	+	302.04	164.01 106.96	16 28	24	4.29
Salbutamol (SALB)	+	240.03	147.96 165.98	20 14	22	2.81
Zilpaterol (ZILP)	+	262.03	202.05 185.01	22 24	22	3.93
Terbutaline hemisulfate (TERB)	+	226.00	152.00 106.97	14 30	26	2.73
Amoxicillin (AMOX)	+	367.07	159.96 90.89	16 40	28	6.43
Ampicillin (AMP)	+	349.97	105.95 159.94	20 14	34	4.97
Benzylpenicillin (BNPC)	+	334.99	90.96 80.94	42 52	44	7.39
Lincomycin (LINK)	+	407.06	126.02 41.75	34 72	22	3.89
Tylosin (TYLS)	+	916.3	173.99 100.88	46 52	74	6.86
Trimethoprim (TRIP)	+	290.97	122.94 229.94	28 24	26	3.81
Cephapirin (CEPR)	+	423.93	291.93 151.89	14 28	42	6.08
Tetracycline (TETC)	+	445.03	410.01 153.90	20 34	40	4.18
Cloxacillin (CLCN)	+	435.94	159.97 276.96	18 14	26	6.94
Oxacillin (OXIN)	+	402.05	159.96 243.03	10 12	24	6.90
Cefalexin (CEFA)	+	347.97	157.86 173.93	8 14	30	2.75
Ceftiofur (CEFT)	+	523.96	241.00 125.17	16 58	34	9.40
Enrofloxacin (ENRO)	+	360.05	245.09 72.02	30 36	36	4.65
Ciprofloxacin (CIPR)	+	332.01	245.05 230.94	40 28	38	4.49
Oxytetracycline (OXTT)	+	462.01	426.02 200.93	38 30	36	4.37
Sulfachloropyridazine (SUPZ)	+	284.90	155.93 91.93	16 34	28	3.93
Sulfadiazine (SUDI)	+	250.97	91.93 155.93	30 14	28	2.49
Sulfadimethoxine (SUDM)	+	310.97	155.93 91.93	20 32	36	5.41
Sulfadimidine (SULD)	+	278.95	185.93 91.93	18 36	34	3.57
Sulfamethoxazole (SULM)	+	253.91	92.00 155.94	30 16	28	4.07
Carbofuran (CRL)	+	222.1	165.0 123.0	12 22	32	6.39
Carbaryl (CRB)	+	202.0	145.05 127.0	10 32	26	5.93
Parathion (PTN)	+	292.0	210.0 180.0	12 26	30	6.86
Malathion (MTN)	+	331.1	98.93 127.0	14 26	30	3.85
Diazinon (DNN)	+	304.97	168.94 153.00	24 24	44	8.18
Dimethoate (DIM)	+	229.90	198.83 124.84	10 20	30	4.77
Atrazine (ATRZ)	+	216.0	174.22 104.14	15 30	32	6.98
Permethrin (PEMT)	+	390.97	355.02 182.92	6 12	34	6.35
Cypermethrin (CIRM)	+	433.0	192.80 90.93	20 12	28	8.07
Deltamethrin (DELM)	+	229.84	198.83 124.85	30 14	30	4.50
Coumaphos (COU)	+	362.90	226.86 306.86	26 18	52	7.75
Dichlorophos (DIRP)	+	220.78	108.89 78.83	20 30	44	6.13
Chlorpyrifos (CHRS)	+	351.78	96.79 199.77	32 16	38	7.47
Fenvalerate (FERT)	+	419.97	166.89 124.88	14 42	38	7.17
Boscalid (BOS)	+	342.94	306.94 139.85	20 20	56	7.46
Fenthoate (FETE)	+	320.86	162.87 246.84	12 12	28	7.31
Fenthion (FEON)	+	278.82	168.87 104.86	18 28	38	7.98
Monocrotophos (MOCR)	+	223.16	192.87 97.83	8 12	30	3.65
Malaoxon (MAON)	+	314.94	126.84 98.80	1426	38	6.79
Methamidophos (MEDF)	+	141.78	93.80 46.82	14 24	38	1.69
Methacrifos (MECF)	+	240.93	208.83 124.83	8 20	32	8.59
Amitraz (AMRZ)	+	294.05	162.96 121.91	14 32	30	7.40
Omethoate (OMAT)	+	213.84	182.82 154.84	12 18	32	2.18
Vamidothion (VAON)	+	287.78	145.92 117.87	14 24	30	4.89
Phosmet (FOST)	+	320.86	246.84 162.87	14 58	32	7.20
Heptenophos (HEPH)	+	250.78	126.83 89.04	16 34	42	7.10
Bifenthrin (BFNT)	+	440.03	180.96 165.87	22 42	24	9.20
Methomyl (MEML)	+	162.84	87.88 105.90	8 10	30	3.29
Zearalenone (ZEAN)	-	316.97	130.87 174.91	30 26	62	6.85
Ochratoxin A (OTAA)	+	404.03	238.92 101.80	30 10	46	7.62
19–17 β Nortestosterone D3 (19ND3)	+	278.10	108.95	26	46	7.26
Clenbuterol-d6 (CLEND6)	+	283.03	203.56	16	22	4.89
Brombuterol-d9 (BROMD9)	+	375.93	293.87	18	24	5.45
Mabuterol-d9 (MABTD9)	+	320.07	237.94	18	24	5.50
Clenpenterol-d5 (CLEPD5)	+	296.00	203.10	16	24	5.71
Isosuxprin-d5 hemifumarate (ISOXD5)	+	308.15	120.95	16	26	4.37
Cimbuterol-d9 (CIMBD9)	+	243.07	160.96	16	20	3.25
Ractopamine-d6 (RACTD6)	+	308.10	168.05	16	24	4.33
Salbutamol-d9 (SALBD9)	+	249.08	148.59	20	24	2.81
Zilpaterol-d7 (ZILPD7)	+	269.08	185.15	24	22	3.93
Terbutalin-d9 (TERBD9)	+	235.07	152.83	16	34	5.52
Flunixin–3 (FLUXD3)	+	300.03	263.98	36	28	7.74
Penicillin G–d7 (PENGD7)	+	374.03	159.94	16	32	6.39
β-zearalenol-d4 (ZEAND4)	-	323.03	160.02	30	68	7.20
Carbofuran-d3 (CARBD3)	+	224.97	164.91	12	22	6.46
Atrazine-d5 (ATRZD5)	+	220.98	100.90	24	34	7.02
Chlorpyrifos-d10 (CHRD10)	+	361.82	98.74	30	26	6.90

### Sample preparation

Six extraction protocols during the optimization of the extraction method were investigated for extraction of the 72 compounds from urine. In two extraction protocols liquid-liquid extraction (LLE) was applied, while solid phase extraction (SPE) was applied to the remaining four protocols. This characterization was as follows: LLE without enzymatic hydrolysis (protocol 1); LLE with enzymatic hydrolysis (protocol 2); SPE using OASIS HLB cartridges without enzymatic hydrolysis (protocol 3); SPE using OASIS HLB cartridges with enzymatic hydrolysis (protocol 4); SPE using DSC-MCAX cartridges without enzymatic hydrolysis (protocol 5) and lastly SPE using DSC-MCAX cartridges with enzymatic hydrolysis (protocol 6). Enzymatic hydrolysis was performed with β-glucuronidase aryl sulfatase (Helix pomatia). Sample preparation steps for all protocols are given below. The initial preparation of the samples prior to extraction, as well as the spiking of urine samples with standards and internal standards are the same for all protocols.

#### All protocols

In the first step, 30 ml urine sample was centrifuged 5 min, at 2000 rpm and room temperature. Тhis step was used to remove proteins from the matrix. After centrifugation, 5 ml of urine sample was fortified with the standards and internal standards. Prior to extraction the samples were left to stand for 10 min. Next, the samples were prepared with different protocols, as follows.

#### Protocol 1

5 ml of 0.2 M sodium acetate buffer (pH = 5) and 5 ml 0.02 M Phosphate buffer (PBS) (pH = 7.2) (1:1, v/v) were added to the samples, and the samples were shaken for 1 min on a vortex. After this step samples were centrifuged 5 min, at 2000 rpm, at room temperature followed by LLE. 10 ml methanol:acetonitrile:acetic acid (49:49:2, v/v/v) was added and the samples were shaken for 1 min on a vortex and centrifuged again for 5 min, at 2000 rpm and room temperature and the supernatant was transferred to new test tubes. Тhe LLE was repeated with 10 ml of ethyl acetate: hexane (40:60, v/v). The samples were shaken for 1 min on a vortex and centrifuged 5 min, at 2000 rpm and room temperature. Тhe supernatant was collected, added to the first supernatant and the mixture was evaporated under nitrogen to near dryness at 35 °C. The residue was reconstituted with 1 ml of the mobile phase (95:5, v/v, Mobile phase A: Mobile phase B), filtered through a 0.45 μm membrane filter into 2 ml autosampler vials and analysed on the LC-MS/MS after separation on a reverse phase column.

#### Protocol 2

5 ml of 0.2 M sodium acetate buffer (pH = 5) and 5 ml 0.02 M phosphate buffer (PBS) (pH = 7.2) (1:1, v/v) were added to the samples, and the samples were shaken for 1 min on a vortex. Further, 20 µl of β-glucuronidase aryl sulfatase was added and the samples were incubated for 17 h at 37 °C. After cooling to room temperature, the samples were centrifuged for 5 min, at 2000 rpm and room temperature, followed by LLE. The LLE and the next steps for sample preparation were the same as in protocol 1.

#### Protocol 3

5 ml of 0.2 M sodium acetate buffer (pH = 5) and 5 ml 0.02 M phosphate buffer (PBS, pH = 7.2) were added and the samples were shaken for 1 min on a vortex before spinning of a centrifuge for 5 min at 2000 rpm and room temperature. This was followed SPE extraction. Oasis HLB cartridges were activated and conditioned with 5 ml of methanol and 5 ml of water. The extract was passed through the cartridges at one drop per second and the cartridge dried, washed with 5 ml of water and dried again. The residues were eluted first with 4 ml of methanol:acetonitrile:ammonium hydroxide (48.5:48.5:3, v/v/v) and then with 4 ml of methanol: dichloromethane (1.5:8.5, v/v). The eluent was evaporated to dryness at 35 °C under nitrogen, the residues were reconstituted with 1 ml of the mobile phase (Mobile phase A: Mobile phase B, 95:5, v/v, B) pressed through 0.45 μm membrane filter into 2 ml autosampler vials prior to LC–MS/MS analysis after separation on a C-18 column.

#### Protocol 4

5 ml of 0.2 M sodium acetate buffer (pH = 5) and 5 ml 0.02 M Phosphate buffer (PBS, pH = 7.2) were added and the samples were shaken for 1 min on a vortex before spinning of a centrifuge for 5 min at 2000 rpm and room temperature. In the next step, βeta-glucuronidase aryl sulfatase (20 µl) was added and the samples incubated for 17 h at 37 °C followed after cooling, by centrifugation for 5 min at 2000 rpm and room temperature. Further, the same approach was applied as well as in protocol 3.

#### Protocol 5

In this protocol, DSC-MCAX cartridges instead of Oasis HLB cartridges were used and the rest of the conditions remaining the same as in the protocol 3.

#### Protocol 6

In this protocol, Oasis HLB cartridges were replaced by DSC-MCAX cartridges and the rest of the conditions remaining the same as in the protocol 6.

The sample preparation steps for all protocols are given in the Table 4.

**Table 4:** Recovery in bovine urine samples for each protocol

Analytes	Added concen-tration (µg/ L)	LLE* without EH** (%)	LLE with EH	SPE*** with Oasis HLB cartridges without EH (%)	SPE with Oasis HLB cartridges (%) with EH	SPE with DSC-MCAX cartridges (%) without EH	SPE with DSC-MCAX cartridges (%) with EH
Thiouracil (TU)	5 10 15	nd****	nd	88.6 92.4 94.1	93.0 101.1 101.5	65.8 62.4 55.7	70.4 75.2 77.8
Methylthiouracil (MTU)	5 10 15	nd	nd	80.3 85.3 93.5	95.6 94.6 96.1	72.4 77.4 75.6	79.8 80.1 84.3
Propylthiouracil (PTU)	5 10 15	nd	nd	70.2 73.1 77.5	77.0 84.5 89.9	67.8 66.4 60.2	71.4 74.2 77.4
Tapazole (TAP)	5 10 15	nd	nd	73.5 71.5 79.9	86.4 84.8 93.9	74.7 72.3 78.5	81.4 77.8 79.3
Testosterone (TEST)	7.5 15 22.5	65.6 60.2 61.3	69.6 65.4 64.3	74.2 73.5 70.0	87.2 88.2 90.5	69.7 74.3 72.1	80.4 76.3 71.2
Methyltestosterone (MEST)	0.25 0.50 0.75	48.9 45.3 50.2	58.9 59.0 52.4	65.2 60.1 67.7	86.0 91.0 94.8	60.2 61.3 64.7	67.8 71.2 65.1
Boldenone (BOLD)	0.5 1.0 1.5	51.3 54.8 60.2	55.4 59.7 61.6	63.4 71.2 60.1	84.8 104.2 91.6	54.6 52.2 58.3	64.5 60.2 67.4
19 Nortestosterone (19 NO)	0.25 0.5 0.75	44.6 40.2 51.3	60.1 62.2 57.7	55.4 61.2 63.1	84.4 94.2 95.2	65.4 60.7 59.2	70.4 66.3 67.1
Stanozolol (STZL)	0.25 0.5 0.75	65.6 60.2 63.8	69.6 66.7 70.2	70.0 59.4 63.1	88.8 91.4 94.8	71.3 69.2 61.3	75.6 71.3 69.4
Clostebol (CLBL)	7.5 15 22.5	39.4 35.5 41.3	52.5 54.8 50.3	71.2 62.4 60.1	89.6 93.6 93.0	57.6 54.3 61.8	65.6 69.7 66.3
Zeranol (ZENL)	0.5 1.0 1.5	49.7 42.5 44.5	60.3 61.6 54.8	54.1 55.2 61.1	79.2 81.6 89.9	67.3 70.5 62.4	71.3 72.5 79.2
Taleranol (TANL)	0.5 1.0 1.5	55.2 51.1 57.8	64.1 63.2 58.8	58.4 67.8 71.3	87.2 90.8 81.9	61.6 67.3 70.2	78.2 69.1 66.7
Clenbuterol (CLEN)	0.05 0.1 0.15	41.6 43.1 47.8	64.3 56.4 60.0	49.4 54.1 61.2	74.0 79.0 88.0	56.5 59.2 55.4	70.3 71.2 65.6
Brombuterol (BROM)	0.05 0.1 0.15	51.4 50.8 57.2	61.3 65.4 63.2	64.3 69.1 58.3	76.0 86.0 78.0	66.3 67.1 75.4	69.4 77.6 80.2
Mabuterol (MABT)	0.05 0.1 0.15	35.4 32.1 40.2	47.8 45.2 43.4	51.3 49.1 53.5	84.0 71.0 75.3	57.4 59.2 51.5	60.2 59.9 54.3
Clenpenterol (CLEP)	0.05 0.1 0.15	46.8 49.2 50.1	54.7 53.2 64.5	61.6 54.1 61.3	96.0 113.0 91.3	66.3 69.2 71.8	75.6 70.2 80.5
Isoxuprin (ISOX)	0.25 0.50 0.75	51.2 47.8 55.7	60.8 58.9 53.4	71.4 75.3 81.2	90.8 102.8 98.4	54.6 50.2 59.8	66.3 57.8 70.2
Cimbuterol (CIMB)	0.05 0.1 0.15	40.8 45.6 44.7	60.2 55.7 61.3	54.5 61.3 64.1	74.0 117.0 88.0	59.6 64.3 71.8	62.4 75.4 72.1
Ractopamine (RACT)	0.25 0.50 0.75	42.4 45.0 51.2	60.8 63.2 59.5	46.1 49.7 59.4	95.2 91.4 93.0	63.2 66.4 71.3	77.4 70.5 73.2
Salbutamol (SALB)	0.25 0.50 0.75	61.5 64.2 59.4	69.7 66.4 67.8	69.7 66.3 60.1	78.8 97.4 85.9	74.6 70.2 79.4	83.1 75.6 88.1
Zilpaterol (ZILP)	0.25 0.50 0.75	39.4 33.5 37.8	51.2 43.1 58.7	66.3 59.7 63.1	85.2 103.4 102.1	55.4 59.2 53.1	63.8 70.2 61.5
Terbutaline (Terb)	0.25 0.50 0.75	45.4 55.2 53.4	59.8 59.9 62.3	47.3 51.3 55.1	77.6 103.8 88.8	65.4 60.2 59.8	70.2 75.4 77.9
Amoxicillin (AMOX)	7.5 15 22.5	62.5 63.8 57.6	70.0 65.4 61.8	64.8 63.0 59.3	96.0 94.0 85.8	63.5 66.8 71.3	78.2 81.3 71.5
Ampicillin (AMP)	7.5 15 22.5	44.6 50.2 45.3	54.8 55.9 49.3	74.2 73.8 85.4	92.0 98.2 94.7	69.4 65.6 60.2	81.4 75.6 78.3
Benzylpenicillin (BNPC)	7.5 15 22.5	39.0 41.5 46.0	57.0 54.2 61.3	84.3 81.5 75.2	101.3 101.3 83.4	64.6 69.2 60.4	74.5 78.8 77.3
Lincomycin (LINK)	7.5 15 22.5	36.5 42.8 44.6	51.8 52.3 54.6	78.2 85.3 74.6	89.5 82.6 92.1	75.5 74.1 79.2	81.3 77.4 78.2
Tylosin (TYLS)	7.5 15 22.5	55.8 52.1 59.3	62.4 60.2 67.8	82.2 87.5 91.3	90.7 88.0 92.6	61.2 67.4 70.2	75.5 79.8 74.6
Trimethoprim (TRIP)	7.5 15 22.5	44.6 47.5 54.3	59.2 54.7 60.2	84.1 91.2 87.3	94.4 93.3 83.1	59.8 55.3 56.2	75.6 77.8 71.4
Cephapirin (CEPR)	7.5 15 22.5	55.4 55.3 61.4	63.4 65.8 60.2	74.1 81.3 83.2	92.0 90.4 96.5	66.3 65.2 70.4	77.8 74.5 71.3
Tetracycline (TETC)	7.5 15 22.5	50.2 51.0 47.6	61.3 59.8 59.3	84.5 79.4 83.8	86.8 91.3 89.6	64.5 60.2 70.1	73.4 75.2 80.4
Cloxacillin (CLCN)	7.5 15 22.5	33.6 39.8 34.2	49.8 52.3 47.8	75.2 81.3 86.8	92.4 86.7 83.5	74.3 71.8 88.2	79.4 72.3 89.5
Oxacillin (OXIN)	7.5 15 22.5	39.0 43.5 41.0	55.6 60.2 51.3	77.8 71.2 83.4	88.4 92.1 102.7	66.3 60.2 69.7	74.2 77.1 74.3
Cefalexin (CEFA)	7.5 15 22.5	36.5 41.3 47.8	50.2 44.6 47.2	80.2 75.3 78.8	101.3 108.8 97.3	60.3 61.5 69.2	62.4 66.4 67.1
Ceftiofur (CEFT)	7.5 15 22.5	45.2 41.3 46.2	55.6 59.3 51.8	69.5 65.4 71.4	81.3 92.1 81.8	54.3 59.2 60.1	71.3 75.6 77.9
Enrofloxacin (ENRO)	7.5 15 22.5	55.8 62.3 61.5	56.8 59.4 64.5	90.7 84.4 85.2	89.4 88.9 85.3	67.8 72.3 75.6	80.4 79.2 81.3
Ciprofloxacin (CIPR)	7.5 15 22.5	60.0 64.8 58.2	65.2 64.3 61.8	81.3 88.7 90.4	93.4 94.2 82.7	64.6 68.2 70.2	74.6 77.2 70.5
Oxytetracycline (OXTT)	7.5 15 22.5	53.5 59.6 48.2	63.2 61.7 66.3	75.5 72.1 78.3	80.2 88.4 81.8	71.3 77.6 70.2	75.6 77.8 73.2
Sulfachloropyridazine (SUPZ)	7.5 15 22.5	55.4 56.2 60.2	65.7 69.2 63.8	88.2 84.6 91.3	84.2 84.0 86.2	71.3 77.6 69.2	79.8 80.7 74.3
Sulfadiazine (SUDI)	7.5 15 22.5	47.8 44.3 51.2	60.4 65.2 63.5	88.4 81.0 82.7	90.7 90.0 103.5	66.5 60.2 61.3	69.2 70.1 75.6
Sulfadimethoxine (SUDM)	7.5 15 22.5	50.2 53.8 55.6	59.2 57.6 61.3	92.4 91.7 85.2	82.6 85.3 82.7	71.3 66.2 69.7	77.6 74.3 71.2
Sulfadimidine (SULD)	7.5 15 22.5	47.5 43.2 51.7	55.4 52.3 57.9	97.6 91.3 95.5	98.7 90.2 83.5	55.8 59.2 57.3	61.3 62.4 66.6
Sulfamethoxazole (SULM)	7.5 15 22.5	55.2 59.6 50.1	60.2 64.5 62.0	87.4 80.2 83.5	98.7 93.3 104.1	64.5 69.1 60.2	65.4 73.5 77.2
Carbofuran (CRL)	7.5 15 22.5	60.1 54.3 55.2	65.6 60.2 63.2	86.3 81.2 91.5	90.6 88.4 93.3	74.3 70.8 78.5	81.5 79.8 79.4
Carbaryl (CRB)	7.5 15 22.5	47.8 52.6 55.3	65.7 66.4 60.2	75.5 71.3 72.1	94.6 96.0 90.2	60.2 57.6 53.8	65.6 70.9 73.5
Parathion (PTN)	7.5 15 22.5	35.8 39.2 37.4	50.2 53.5 51.4	69.6 74.5 71.8	89.3 92.0 87.6	66.2 70.4 74.2	80.4 75.2 76.3
Malathion (MTN)	7.5 15 22.5	37.7 36.2 43.5	55.6 39.8 47.5	66.5 69.4 60.2	101.3 98.2 107.1	60.4 60.2 67.4	70.3 65.6 68.4
Diazinon (DNN)	7.5 15 22.5	44.6 51.8 50.2	55.4 56.3 49.7	82.4 87.5 79.8	88.0 85.3 85.7	66.3 61.2 63.4	71.4 72.5 79.4
Dimethoate (DIM)	7.5 15 22.5	46.3 47.5 44.2	60.3 64.2 57.4	88.6 82.3 84.4	92.1 92.0 94.2	59.8 54.3 57.5	70.2 71.8 66.4
Atrazine (ATRZ)	7.5 15 22.5	51.6 57.8 53.6	60.3 66.4 67.5	75.5 71.3 82.8	96.0 91.3 89.3	64.6 67.8 69.5	74.5 78.2 81.2
Permethrin (PEMT)	7.5 15 22.5	50.4 51.3 47.6	61.3 55.4 60.2	80.5 81.3 87.8	96.0 94.0 89.8	65.6 61.3 67.2	69.5 69.1 68.4
Cypermethrin (CIRM)	7.5 15 22.5	44.6 45.2 54.1	67.4 61.3 61.0	80.2 80.8 75.4	85.3 95.4 87.6	55.6 60.2 63.8	71.4 75.2 71.3
Deltamethrin (DELM)	7.5 15 22.5	61.5 57.4 55.3	69.8 71.3 70.0	77.1 72.5 80.6	90.7 89.3 87.6	62.1 66.4 60.8	63.1 67.4 69.2
Coumaphos (COU)	7.5 15 22.5	47.8 52.7 54.6	66.3 61.2 60.4	82.4 87.5 92.4	92.0 86.3 83.1	55.4 59.2 61.5	66.4 69.1 65.6
Dichlorvos (DIRP)	7.5 15 22.5	51.5 54.2 57.8	67.4 59.2 57.1	78.4 71.3 67.5	96.2 87.3 88.1	66.4 61.3 63.8	71.5 75.4 72.3
Chlorpyrifos (CHRS)	7.5 15 22.5	46.6 45.2 40.2	55.2 50.7 51.6	67.8 74.3 81.4	84.1 86.3 89.8	59.8 54.6 57.3	71.4 72.7 79.2
Fenvalerate (FERT)	7.5 15 22.5	43.2 43.6 49.5	50.4 54.3 57.8	84.6 80.2 87.4	90.6 87.7 92.4	60.2 64.5 66.1	75.4 70.2 71.3
Boscalid (BOS)	7.5 15 22.5	51.5 54.6 52.2	60.4 66.4 62.8	90.4 86.6 87.1	93.3 92.0 81.2	78.2 70.4 73.3	80.1 85.5 74.3
Phenthoate (FETE)	7.5 15 22.5	49.5 43.5 52.5	55.7 55.9 50.3	77.1 70.2 79.4	85.3 97.3 103.1	65.1 60.8 67.2	69.3 77.5 72.1
Fenthion (FEON)	7.5 15 22.5	39.5 43.5 47.2	51.7 54.3 60.0	84.5 77.8 81.3	92.0 92.1 86.2	74.5 70.2 72.3	78.4 72.3 79.5
Monocrotophos (MOCR)	7.5 15 22.5	53.5 59.8 55.5	64.6 64.0 59.8	87.5 84.4 91.7	94.6 84.0 83.1	66.3 64.2 67.1	69.7 64.6 69.2
Malaoxon (MAON)	7.5 15 22.5	55.0 50.1 54.3	61.0 61.0 64.6	85.6 81.7 91.3	97.3 90.67 94.67	54.7 59.6 61.3	71.4 74.4 69.7
Methamidophos (MEDF)	7.5 15 22.5	49.5 47.6 42.1	51.3 49.2 47.8	80.2 84.4 80.8	102.6 90.1 88.4	55.7 59.2 57.4	65.6 69.5 64.1
Metacrifos (MECF)	7.5 15 22.5	60.2 58.5 66.4	69.2 64.7 67.1	87.6 78.2 80.3	101.3 92.0 88.4	64.7 69.2 71.3	74.5 71.5 77.8
Amitraz (AMRZ)	7.5 15 22.5	39.8 43.5 47.8	48.5 47.4 51.2	84.6 87.8 93.5	93.3 94.0 85.3	54.3 59.6 61.4	70.4 74.4 79.2
Omethoate (OMAT)	7.5 15 22.5	44.1 47.8 41.3	51.3 55.6 60.3	82.2 87.4 88.6	92.0 97.3 99.6	70.4 64.6 67.3	80.5 74.6 79.8
Vamidothion (VAON)	7.5 15 22.5	56.6 60.3 54.2	60.2 51.3 57.2	74.6 74.8 81.4	85.3 92.1 84.0	59.6 64.8 69.7	64.6 73.2 70.4
Phosmet (FOST)	7.5 15 22.5	55.7 59.6 49.8	60.4 60.8 63.5	84.6 80.7 85.3	96.0 89.3 93.3	70.5 64.7 66.8	71.8 69.6 74.2
Heptenophos (HEPH)	7.5 15 22.5	50.2 57.6 61.4	57.8 58.2 67.4	69.7 65.4 74.7	93.3 94.0 86.2	58.8 56.4 63.8	66.6 69.2 64.6
Bifenthrin (BFNT)	7.5 15 22.5	40.5 46.2 47.8	51.2 53.2 57.4	80.7 85.4 79.9	85.3 88.7 87.5	66.4 70.5 74.5	81.5 79.3 78.2
Methomyl (MEML)	7.5 15 22.5	50.6 53.2 57.8	63.2 66.1 66.2	84.8 90.2 82.4	104.0 99.3 85.78	70.2 75.5 71.3	80.5 79.5 72.8
Zearalenone (ZEAN)	7.5 15 22.5	53.5 54.2 47.8	60.8 67.5 62.1	92.5 95.7 99.4	89.3 84.0 92.4	77.8 72.5 70.4	81.2 74.6 79.7
Ochratoxin A (OTAA)	7.5 15 22.5	44.6 49.2 49.0	53.8 55.1 61.3	88.5 91.3 80.8	98.7 86.0 88.4	66.5 61.2 69.3	77.8 76.4 72.4

* LLE – liquid-liquid extraction, ** EH - enzymatic hydrolyze, *** SPE – solid-phase extraction, **** nd - not detected

### LC-MS/MS analysis

The LC-MS/MS (Waters ACQUITY™ Ultra Performance LC coupled to Waters ACQUITY™ TOQ, Milford, MA, USA) used for identification and quantification of the analytes was equipped with a binary pump, vacuum degasser, thermostated autosampler, thermostated column manager. A Kinetex C18 LC column (50 × 2.1 mm, 2.6 μm, Phenomenex, Torrance, CA, USA) was used for chromatographic separation. MassLynx version 4.1 software (Waters, Milford, MA, USA) was used for instrument control, data acquisition and processing of results.

The LC conditions were as follow: mobile phase flow rate: 0.2 ml/min; column temperature: 40 °C, elution program: 0–1 min, 95 − 80% A; 1–4 min, 80 − 60% A; 4–8 min, 60–95% A; 8–12 min, 95% A; mobile phase A contains: water with 5 mM ammonium acetate, 0.01% formic acid and 0.01% trichloroacetic acid; mobile phase B contains: methanol with 0.1% formic acid, oven temperature: 4 °C; injection volume: 5 µl. The MS/MS conditions were optimized as follows: capillary voltage of 3.0 kV; source temperature of 150 °C; desolvation temperature of 400 °C; cone gas at 100 L/h; desolvation gas at 300 L/h. Both positive and negative electrospray ionization were used along with multiple reaction monitoring (MRM) [10, 26].

### Validation study

Validation of the method was performed according to the European Commission Regulation 2021/808 [25]. The parameters evaluated were: linearity, limit of detection (LOD), limit of quantification (LOQ), decision limit (CCα), detection capability (CCβ), accuracy determined by estimating trueness (recovery), precision (repeatability and reproducibility) and stability.

Matrix-matched calibration curves were prepared to determine the linearity. The blank urine samples were fortified at six concentration levels (for group 1: 2.0; 10.0; 25.0; 50.0 75.0 and 100 µg/ L; for group 2: 0.25; 1.0; 2.5; 5.0; 7.5 and 10.0 µg/ L; for group 3: 0.05; 1.0; 2.5; 5.0; 7.5 and 10.0 µg/ L; for group 4: 0.5; 1.0; 2.5; 5.0; 7.5 and 10 µg/ L; for group 5: 5.0; 10.0; 25.0; 50.0; 75.0 and 100.0 µg/ L). The calibration curves were prepared every day with each series of analyzed samples, each concentration level performed injected in triplicate. The coefficient of correlation (R^2^) was calculated by least squares linear regression analysis.

Decision limit (CCα). The CCα means the concentration of target analyte at and above which it can be concluded with an error probability of α (α = 1%) that a sample is non-compliant and the value 1 – α means statistical certainty in percentage that the permitted limit has been exceeded. CCα was calculated according to 2021/808/EC [25].

Detection capability (CCβ). The CCβ means the smallest concentration of the target analyte that may be detected or quantified in a sample with an error probability of β (β = 5%). CCβ was calculated according to 2021/808/EC [25].

The LOD and LOQ were determined as the lowest spiked concentration evaluated that gave a signal to noise (S/N) ratio of 3:1 for LOD and 10:1 for LOQ [28–30].

The intraday (single day, n = 6) and interday (three consecutive days, n = 18) recovery values were calculated at three spiking levels (x0.5/ x1, and x1.5 the MMPR for substances with MMPR; while for substances without MMPRs and maximum residue limits (MRLs), the samples were fortified according to the sensitivity of the method) (Tables 5 and 6, respectively). Repeatability and reproducibility, in terms of the CV (coefficient of variation for repeatability (CV_r_) and coefficient of variation for reproducibility (CV_R_)) of precision were estimated with spiking of the urine samples with standards and internal standards at the same concentration levels as well as recovery. The spiking levels are given in Tables 5 and 6. For reproducibility, the procedure was performed for three consecutive days by three analysts using different batches of blank material, solvents and reagents on different days.

**Table 5:** Linearity of the method

Analytes	Calibration range (µg/L)	R^2^
Thiouracil (TU)	2.0-100.0	0.997
Methylthiouracil (MTU)	2.0-100.0	0.991
Propylthiouracil (PTU)	2.0-100.0	0.997
Tapazole (TAP)	2.0-100.0	0.994
Testosterone (TEST)	5.0-100.0	0.992
Methyltestosterone (MEST)	0.25-10.0	0.993
Boldenone (BOLD)	0.5–10.0	0.995
19 Nortestosterone (19 NO)	0.25-10.0	0.994
Stanozolol (STZL)	0.25-10.0	0.994
Clostebol (CLBL)	5.0-100.0	0.993
Zeranol (ZENL)	0.5–10.0	0.992
Taleranol (TANL)	0.5–10.0	0.992
Clenbuterol (CLEN)	0.05-10.0	0.993
Brombuterol (BROM)	0.05-10.0	0.993
Mabuterol (MABT)	0.05-10.0	0.992
Clenpenterol (CLEP)	0.05-10.0	0.994
Isoxuprin (ISOX)	0.25-10.0	0.994
Cimbuterol (CIMB)	0.05-10.0	0.995
Ractopamine (RACT)	0.25-10.0	0.992
Salbutamol (SALB)	0.25-10.0	0.992
Zilpaterol HCl (ZILP)	0.25-10.0	0.993
Terbutaline (TERB)	0.25-10.0	0.994
Amoxicillin (AMOX)	5.0-100.0	0.995
Ampicillin (AMP)	5.0-100.0	0.992
Benzylpenicillin (BNPC)	5.0-100.0	0.998
Lincomycin (LINK)	5.0-100.0	0.994
Tylosin (TYLS)	5.0-100.0	0.994
Trimethoprim (TRIP)	5.0-100.0	0.992
Cephapirin (CEPR)	5.0-100.0	0.993
Tetracycline (TETC)	5.0-100.0	0.993
Cloxacillin (CLCN)	5.0-100.0	0.996
Oxacillin (OXIN)	5.0-100.0	0.997
Cefalexin (CEFA)	5.0-100.0	0.997
Ceftiofur (CEFT)	5.0-100.0	0.996
Enrofloxacin (ENRO)	5.0-100.0	0.998
Ciprofloxacin (CIPR)	5.0-100.0	0.992
Oxytetracycline (OXTT)	5.0-100.0	0.995
Sulfachloropyridazine (SUPZ)	5.0-100.0	0.998
Sulfadiazine (SUDI)	5.0-100.0	0.997
Sulfadimethoxine (SUDM)	5.0-100.0	0.998
Sulfadimidine (SULD)	5.0-100.0	0.999
Sulfamethoxazole (SULM)	5.0-100.0	0.995
Carbofuran (CRL)	5.0-100.0	0.998
Carbaryl (CRB)	5.0-100.0	0.994
Parathion (PTN)	5.0-100.0	0.994
Malathion (MTN)	5.0-100.0	0.994
Diazinon (DNN)	5.0-100.0	0.994
Dimethoate (DIM)	5.0-100.0	0.993
Atrazine (ATRZ)	5.0-100.0	0.997
Permethrin (PEMT)	5.0-100.0	0.993
Cypermethrin (CIRM)	5.0-100.0	0.997
Deltamethrin (DELM)	5.0-100.0	0.997
Coumaphos (COU)	5.0-100.0	0.994
Dichlorophos (DIRP)	5.0-100.0	0.994
Chloropyrifos (CHRS	5.0-100.0	0.993
Fenvalerate (FERT)	5.0-100.0	0.991
Boscalid (BOS)	5.0-100.0	0.997
Fenthoate (FETE)	5.0-100.0	0.996
Fenthion (FEON)	5.0-100.0	0.992
Monocrotophos (MOCR)	5.0-100.0	0.997
Malaoxon (MAON)	5.0-100.0	0.998
Methamidophos (MEDF)	5.0-100.0	0.995
Metacrifos (MECF)	5.0-100.0	0.993
Amitraz (AMRZ)	5.0-100.0	0.994
Omethoate (OMAT)	5.0-100.0	0.995
Vamidothione (VAON)	5.0-100.0	0.995
Phosmet (FOST)	5.0-100.0	0.999
Heptenophos (HEPH)	5.0-100.0	0.998
Bifenthrin (BFNT)	5.0-100.0	0.998
Methomyl (MEML)	5.0-100.0	0.996
Zearalenone (ZEAN)	5.0-100.0	0.998
Ochratoxin A (OTAA)	5.0-100.0	0.999

**Table 6:** Decision limit, detection capability, limit of detection, limit of quantification of the method and used internal standards

Analytes	CCα* (µg/L)	CCβ** (µg/L)	LOD*** (µg/L)	LOQ**** (µg/L)	Internal standards
Thiouracil (TU)	5.17	8.46	2.03	5.78	/
Methylthiouracil (MTU)	2.26	4.88	1.04	3.56	/
Propylthiouracil (PTU)	2.23	5.22	1.48	4.75	/
Tapazole (TAP)	5.03	8.42	2.22	5.60	/
Testosterone (TEST)	4.87	6.36	1.88	5.45	/
Methyltestosterone (MEST)	0.27	0.48	0.12	0.40	/
Boldenone (BOLD)	0.69	0.95	0.31	0.95	/
19 Nortestosterone (19 NO)	0.25	0.48	0.15	0.47	19 − 17β Nortestosteron-d3
Stanozolol (STZL)	0.34	0.50	0.14	0.38	/
Clostebol (CLBL)	4.36	8.02	1.56	4.88	/
Zeranol (ZENL)	0.62	0.93	0.26	0.89	/
Taleranol (TANL)	0.58	0.87	0.19	0.94	/
Clenbuterol (CLEN)	0.06	0.09	0.02	0.08	Clenbuterol-d6
Brombuterol (BROM)	0.07	0.09	0.03	0.08	Brombuterol-d9
Mabuterol (MABT)	0.08	0.10	0.03	0.09	Mabuterol-d9
Clenpenterol (CLEP)	0.06	0.08	0.03	0.09	Clenpenterol-d5
Isoxuprin (ISOX)	0.28	0.38	0.17	0.32	Isoxsuprine-d5
Cimbuterol (CIMB)	0.05	0.10	0.01	0.05	Cimbuterol-d9
Ractopamine (RACT0	0.38	0.49	0.16	0.49	Ractopamine-d6
Salbutamol (SALB)	0.26	0.41	0.17	0.48	Salbutamol-d9
Zilpaterol (ZILP)	0.36	0.49	0.14	0.40	Zilpaterol-d7
Terbutaline (TERB)	0.22	0.43	0.11	0.42	Terbutaline-d9
Amoxicillin (AMOX)	9.25	12.18	2.26	7.03	Penicillin G-d7
Ampicillin (AMP0	8.14	11.25	2.71	7.12	Penicillin G-d7
Benzylpenicillin (BNPC)	10.12	12.18	2.05	6.94	Penicillin G-d7
Lincomycin (LINK)	7.15	9.18	2.16	6.85	/
Tylosin (TYLS)	9.11	13.54	1.36	6.41	/
Trimethoprim (TRIP)	8.82	12.46	1.78	7.15	/
Cephapirin (CEPR)	9.12	11.35	2.54	7.46	/
Tetracycline (TETC)	7.00	9.12	2.01	6.54	/
Cloxacillin (CLCN)	10.54	12.83	2.22	7.10	Penicillin G-d7
Oxacillin (OXIN)	8.15	10.41	1.51	7.52	Penicillin G-d7
Cefalexin (CEFA)	12.11	15.16	2.09	6.57	/
Ceftiofur (CEFT)	7.35	10.12	1.58	6.96	/
Enrofloxacin (ENRO)	9.28	12.75	2.04	7.17	Flunixin–d3
Ciprofloxacin (CIPR)	8.64	12.00	0.88	4.02	Flunixin–d3
Oxytetracycline (OXTT)	6.28	8.17	2.11	6.78	/
Sulfachloropyridazine (SUPZ)	8.25	10.22	1.56	7.02	/
Sulfadiazine (SUDI)	7.17	9.25	1.14	5.95	/
Sulfadimethoxine (SUDM)	7.38	8.92	2.46	7.26	/
Sulfadimidine (SULD)	11.35	14.80	2.11	6.58	/
Sulfamethoxazole (SULM)	9.18	12.35	1.57	5.92	/
Carbofuran (CRL)	6.11	9.28	2.54	7.35	Carbofuran-d3
Carbaryl (CRB)	8.25	12.41	1.88	6.51	/
Parathion (PTN)	7.15	9.12	0.95	4.12	/
Malathion (MTN)	7.81	10.14	1.36	5.06	/
Diazinon (DNN)	9.52	11.88	1.95	6.71	/
Dimethoate (DIM)	8.35	9.22	2.07	6.52	/
Atrazine (ATRZ)	11.80	13.59	2.38	7.28	Atrazine-d5
Permethrine (PEMT)	9.87	12.35	2.17	7.11	/
Cypermethrine (CIRM)	9.38	12.13	1.41	6.36	/
Deltamethrine (DELM)	11.85	14.45	0.85	2.92	/
Coumaphos (COU)	10.35	13.12	2.68	7.51	/
Dichlorophos (DIRP)	8.81	11.35	1.55	7.25	/
Chlorpyrifos (CHRS)	7.38	9.86	1.47	5.48	Chlorpyrifos-d10
Fenvalerate (FERT)	9.12	10.92	2.42	7.50	/
Boscalid (BOS)	6.54	8.25	1.78	6.28	/
Fenthoate (FETE)	8.78	11.38	1.41	5.11	/
Fenthione (FEON)	7.05	11.06	2.12	6.25	/
Monocrotophos (MOCR)	8.12	11.48	2.30	7.08	/
Malaoxon (MAON)	7.48	9.86	2.67	7.35	/
Methamidophos MEDF	10.15	14.00	1.45	5.00	/
Metacrifos (MECF)	8.15	11.48	2.00	6.28	/
Amitraz (AMRZ)	9.01	10.56	1.42	6.02	/
Omethoate (OMAT)	8.45	11.48	1.88	6.89	/
Vamidothion (VAON)	9.12	12.90	1.56	7.25	/
Phosmet (FOST)	9.41	11.52	1.01	4.11	/
Heptenophos (HEPH)	7.08	11.06	1.47	7.51	/
Bifenthrin (BFNT)	7.35	9.42	2.05	7.35	/
Methomyl (MEML)	10.14	13.78	1.92	5.91	/
Zearalenone (ZEAN)	6.35	10.08	0.98	4.38	β-zearalenol-d4
Ochratoxin A (OTAA)	8.12	11.35	1.14	4.08	/

*CCα - decision limit, **CCβ - detection capability, ***LOD – limit of detection, ****LOQ – limit of quantification

Stability tests are performed for stock standard solutions, working standard solutions and standards in final matrix. Stock standard solutions were tested at -20 °C within 1 year, once per month. Working standard solutions were tested at − 20 °C every week. Stability in the final extract was tested at − 20 °C within 1 week, once per day, at different concentrations.

## Results and discussion

### MS/MS optimization

The MS/MS conditions including collision energy, cone voltage and dwell time (0.025 s), as well as selection of appropriate diagnostic ions are summarized in Table 7. This follows direct infusion of standard and internal standard working solutions (1.0 µg/ml).

**Table 7:** Intraday recovery and repeatability of the method

Analytes	Added concentration (µg/L)	Average concentration in samples (µg/L) (n = 6)	Standard deviation (µg/L)	Recovery (%)	Repeatability (CVr, %)
Thiouracil (TU)	5 10 15	4.65 10.11 15.22	0.25 0.75 1.12	93.0 101.1 101.5	5.38 7.42 7.77
Methylthiouracil (MTU)	5 10 15	4.78 9.46 14.42	0.31 0.62 1.27	95.6 94.6 96.1	6.49 6.55 8.81
Propylthiouracil (PTU)	5 10 15	3.85 8.45 13.48	0.12 0.62 1.01	77.0 84.5 89.9	3.12 7.34 7.49
Tapazole (TAP)	5 10 15	4.32 8.48 14.08	0.28 0.39 0.78	86.4 84.8 93.9	6.48 4.60 5.54
Testosterone (TEST)	7.5 15 22.5	4.36 8.82 13.58	0.38 0.72 1.05	87.20 88.20 90.53	8.72 8.16 7.73
Methyltestosterone (MEST)	0.25 0.50 0.75	0.21 0.46 0.70	0.014 0.041 0.052	86.0 91.0 94.8	6.67 8.91 7.43
Boldenone (BOLD)	0.5 1.0 1.5	0.42 1.04 1.37	0.034 0.079 0.171	84.8 104.2 91.6	8.10 7.60 12.48
19 Nortestosteron (19 NO)	0.25 0.5 0.75	0.21 0.47 0.71	0.011 0.032 0.048	84.4 94.2 95.2	5.24 6.81 6.76
Stanozolol (STZL)	0.25 0.5 0.75	0.22 0.46 0.71	0.017 0.051 0.074	88.8 91.4 94.8	7.28 11.09 10.42
Clostebol (CLBL)	7.5 15 22.5	4.48 9.36 13.95	0.25 1.15 1.66	89.6 93.6 93.0	5.58 12.29 11.90
Zeranol (ZENL)	0.5 1.0 1.5	0.41 0.88 1.27	0.056 0.111 0.068	81.6 88.7 84.8	13.66 12.61 5.35
Taleranol (TANL)	0.5 1.0 1.5	0.45 0.92 1.43	0.038 0.084 0.092	90.8 92.4 95.3	8.44 9.13 6.43
Clenbuterol (CLEN)	0.05 0.1 0.15	0.04 0.08 0.13	0.003 0.004 0.009	74.0 79.0 88.0	6.50 5.25 6.92
Brombuterol (BROM)	0.05 0.1 0.15	0.04 0.09 0.12	0.005 0.008 0.014	76.0 86.0 78.0	11.75 8.67 11.67
Mabuterol (MABT)	0.05 0.1 0.15	0.04 0.07 0.11	0.004 0.007 0.012	84.0 67.0 75.3	8.75 9.71 10.91
Clenpenterol (CLEP)	0.05 0.1 0.15	0.05 0.11 0.14	0.005 0.009 0.010	96.0 113.0 91.3	9.60 7.82 7.30
Isoxuprin (ISOX)	0.25 0.50 0.75	0.22 0.51 0.74	0.027 0.054 0.079	90.8 102.8 98.4	12.27 10.59 10.68
Cimbuterol (CIMB)	0.05 0.1 0.15	0.04 0.12 0.13	0.005 0.008 0.014	74.0 117.0 88.0	11.22 6.42 10.77
Ractopamine (RACT)	0.25 0.50 0.75	0.24 0.46 0.70	0.021 0.052 0.071	95.2 91.4 93.0	8.75 11.30 10.14
Salbutamol (SALB)	0.25 0.50 0.75	0.20 0.49 0.64	0.022 0.057 0.062	78.8 97.4 85.9	11.00 11.63 9.69
Zilpaterol HCl (ZILP)	0.25 0.50 0.75	0.21 0.52 0.77	0.029 0.050 0.065	85.2 103.4 102.1	13.81 9.62 8.44
Terbutaline (TERB)	0.25 0.50 0.75	0.19 0.52 0.67	0.017 0.036 0.078	77.6 103.8 88.8	8.95 6.92 11.64
Amoxicillin (AMOX)	7.5 15 22.5	7.2 14.1 19.3	0.31 1.42 1.74	96.0 94.0 85.8	4.31 10.07 9.02
Ampicillin (AMP)	7.5 15 22.5	6.9 14.7 21.3	0.78 1.13 1.45	92.0 98.2 94.7	11.30 7.69 6.81
Benzylpenicillin (BNPC)	7.5 15 22.5	7.6 15.2 18.7	0.28 1.01 0.75	101.3 101.3 83.4	3.68 6.64 4.01
Lincomycin (LINK)	7.5 15 22.5	6.7 12.4 20.7	0.29 1.43 1.95	89.5 82.6 92.1	4.33 11.53 9.40
Tylosin (TYLS)	7.5 15 22.5	6.8 13.2 20.9	0.48 1.32 1.14	90.7 88.0 92.6	7.06 10.0 8.84
Trimethoprim (TRIP)	7.5 15 22.5	7.1 14.0 18.7	0.65 1.10 2.05	94.4 93.3 83.1	9.15 7.85 10.96
Cephapirin (CEPR)	7.5 15 22.5	6.9 13.5 21.8	0.38 0.75 1.94	92.0 90.4 96.5	5.51 5.56 8.51
Tetracycline (TETC)	7.5 15 22.5	6.5 13.7 20.1	0.11 0.72 0.96	86.8 91.3 89.6	1.69 5.26 4.78
Cloxacillin (CLCN)	7.5 15 22.5	6.9 13.0 18.8	0.25 1.10 1.15	92.4 86.7 83.5	3.62 8.46 6.12
Oxacillin (OXIN)	7.5 15 22.5	6.6 13.8 23.0	0.36 1.22 2.14	88.4 92.1 102.7	5.45 8.84 9.30
Cefalexin (CEFA)	7.5 15 22.5	7.6 16.3 21.9	0.78 1.02 1.73	101.3 108.8 97.3	10.26 6.26 7.90
Ceftiofur (CEFT)	7.5 15 22.5	6.1 13.8 18.4	0.46 1.18 2.13	81.3 92.1 81.8	7.54 8.55 11.58
Enrofloxacin (ENRO)	7.5 15 22.5	6.7 13.8 19.2	0.94 1.45 1.74	89.4 88.9 85.3	14.02 10.50 9.06
Ciprofloxacin (CIPR)	7.5 15 22.5	7.0 14.1 18.6	0.85 1.74 2.04	93.4 94.2 82.7	12.14 12.34 10.97
Oxytetracycline (OXTT)	7.5 15 22.5	6.0 13.2 18.4	0.64 1.11 1.45	80.2 88.4 81.8	10.67 8.41 7.88
Sulfachlorpyridazine (SUPZ)	7.5 15 22.5	6.3 13.7 19.4	0.95 1.12 1.17	84.2 84.0 86.2	15.07 8.17 6.03
Sulfadiazine (SUDI)	7.5 15 22.5	6.8 13.5 21.3	1.14 1.75 1.41	90.7 90.0 103.5	16.76 12.96 6.62
Sulfadimethoxine (SUDM)	7.5 15 22.5	6.2 12.8 18.6	0.61 0.78 1.31	82.6 85.3 82.7	9.84 6.09 7.04
Sulfadimidine (SULD)	7.5 15 22.5	6.9 13.5 18.8	0.65 1.17 1.48	98.7 90.2 83.5	9.42 8.67 7.87
Sulfamethoxazole (SULM)	7.5 15 22.5	7.4 14.0 23.4	1.22 1.78 1.21	98.7 93.3 104.1	16.48 12.71 5.17
Carbofuran (CRL)	7.5 15 22.5	6.8 13.2 21.0	0.35 0.61 1.11	90.6 88.4 93.3	5.15 4.62 5.29
Carbaryl (CRB)	7.5 15 22.5	7.1 14.4 20.3	0.78 1.02 1.12	94.6 96.0 90.2	10.98 7.08 9.31
Parathion (PTN)	7.5 15 22.5	6.7 13.8 19.7	0.78 1.11 1.23	89.3 92.0 87.6	11.64 8.04 6.24
Malathion (MTN)	7.5 15 22.5	7.6 14.7 24.1	0.64 0.92 1.47	101.3 98.2 107.1	8.42 6.26 6.10
Diazinon (DNN)	7.5 15 22.5	6.6 12.8 19.3	0.32 0.75 1.12	88.0 85.3 85.7	4.85 5.86 5.80
Dimethoate (DIM)	7.5 15 22.5	6.9 13.8 21.2	1.01 1.27 1.59	92.1 92.0 94.2	14.63 9.20 7.50
Atrazine (ATRZ)	7.5 15 22.5	7.2 13.7 20.1	0.54 0.75 1.13	96.0 91.3 89.3	7.06 5.48 5.62
Permethrin (PEMT)	7.5 15 22.5	7.2 14.1 20.2	1.02 1.41 1.51	96.0 94.0 89.8	14.16 10.00 7.48
Cypermethrin (CIRM)	7.5 15 22.5	6.4 14.3 19.7	0.78 0.95 1.43	85.3 95.4 87.6	12.19 6.64 7.26
Deltamethrin (DELM)	7.5 15 22.5	6.8 13.4 19.7	0.91 1.32 1.74	90.7 89.3 87.6	13.38 9.85 8.83
Coumaphos (COU)	7.5 15 22.5	6.9 12.9 18.7	0.56 0.92 1.36	92.0 86.3 83.1	8.12 7.13 7.27
Dichlorvos (DIRP)	7.5 15 22.5	7.2 13.1 19.8	0.78 1.45 1.92	96.2 87.3 88.1	10.83 11.07 9.70
Chlorpyrifos (CHRS)	7.5 15 22.5	6.3 12.9 20.2	0.28 1.43 1.21	84.1 86.3 89.8	4.45 11.09 5.99
Fenvalerate (FERT)	7.5 15 22.5	6.8 13.5 20.8	0.34 0.92 1.13	90.6 87.7 92.4	5.00 6.81 5.43
Boscalid (BOS)	7.5 15 22.5	7.0 13.8 18.4	0.46 1.11 1.42	93.3 92.0 81.2	6.57 8.04 7.72
Fenthoate (FETE)	7.5 15 22.5	6.4 14.6 23.2	0.45 1.01 1.41	85.3 97.3 103.1	7.03 6.92 6.08
Fenthion (FEON)	7.5 15 22.5	6.9 13.8 19.4	0.27 0.88 1.02	92.0 92.1 86.2	3.91 6.37 5.26
Monocrotophos (MOCR)	7.5 15 22.5	7.1 12.6 18.7	0.65 0.68 1.05	94.6 84.0 83.1	9.15 5.40 5.61
Malaoxon (MAON)	7.5 15 22.5	7.3 13.6 21.3	1.14 1.25 1.38	97.3 90.67 94.67	15.62 9.19 6.48
Methamidophos (MEDF)	7.5 15 22.5	7.7 13.5 19.8	1.11 1.82 2.01	102.6 90.1 88.4	14.41 13.48 10.15
Metacrifos (MECF)	7.5 15 22.5	7.6 13.8 22.1	0.41 0.95 1.17	101.3 92.0 88.4	5.39 6.88 5.29
Amitraz (AMRZ)	7.5 15 22.5	7.0 14.1 19.2	1.12 1.41 1.52	93.3 94.0 85.3	16.00 10.00 7.92
Omethoate (OMAT)	7.5 15 22.5	6.9 14.6 22.4	0.28 0.71 1.13	92.0 97.3 99.6	4.06 4.86 5.04
Vamidothion (VAON)	7.5 15 22.5	6.4 13.8 18.9	0.95 1.14 1.38	85.3 92.1 84.0	14.84 8.26 7.30
Phosmet (FOST)	7.5 15 22.5	7.2 13.4 21.0	0.65 0.92 1.12	96.0 89.3 93.3	9.02 6.87 5.33
Heptenophos (HEPH)	7.5 15 22.5	7.0 14.1 19.4	0.65 0.95 1.52	93.3 94.0 86.2	9.29 6.74 7.83
Bifenthrin (BFNT)	7.5 15 22.5	6.4 13.3 19.7	0.54 0.68 0.92	85.3 88.7 87.5	8.43 5.11 4.67
Methomyl (MEML)	7.5 15 22.5	7.8 14.9 19.3	1.11 1.48 1.23	104.0 99.3 85.78	14.23 9.93 6.37
Zearalenone (ZEAN)	7.5 15 22.5	6.7 12.6 20.8	0.49 0.61 0.68	89.3 84.0 92.4	7.31 4.84 3.27
Ochratoxin A (OTAA	7.5 15 22.5	7.4 12.9 19.9	0.92 1.45 1.52	98.7 86.0 88.4	12.43 11.24 7.63

### Optimization of the sample preparation

The recovery test results for the different sample preparation protocols are summarized in Table 8. As can be seen, the thyreostats were not detected with the LLE protocols (first and second protocols). The first protocol results (32.1–66.4%) and those from the second protocol (39.8–71.3%) were not satisfactory according to 2021/808/EC [25]. In protocols 3, 5 and 6 most recoveries were not in range described in 2021/808/EC, namely 50 − 120% for fortification concentration ≤ 1 µg/kg, 70 − 120% for > 1 to 10 µg/kg and 80 − 120% for ≥ 10 µg/kg [25]. The best recoveries were obtained using SPE extraction protocols including enzymatic hydrolysis (protocol 4). The recoveries ranged were from 71.0% for mabuterol spiked at the concentration 0.1 µg/L to 117.0% for cimbuterol spiked at the concentration 0.1 µg/L. The protocol 4 was therefore selected for further validation.

**Table 8:** Interday recovery and reproducibility of the method

Analytes	Added concentration (µg/L)	Average concentration in samples (µg/L) (n = 18)	Standard deviation (µg/L)	Recovery (%)	Reproducibi-lity (CV_R_, %)
Thiouracil (TU)	5 10 15	4.88 10.51 14.32	0.41 1.20 1.80	97.6 105.1 95.5	8.32 11.40 12.54
Methylthiouracil (MTU)	5 10 15	4.28 8.85 13.95	0.40 1.02 1.92	85.6 88.5 93.0	9.36 11.55 13.84
Propylthiouracil (PTU)	5 10 15	4.88 10.10 15.14	0.30 1.27 2.11	97.6 101.0 100.9	6.11 12.54 13.95
Tapazole (TAP)	5 10 15	4.23 9.78 13.66	0.39 0.73 1.56	84.6 97.8 91.1	9.22 7.40 11.54
Testosterone (TEST)	7.5 15 22.5	7.02 14.51 21.46	0.87 1.61 2.12	93.6 96.7 95.4	12.41 11.08 9.87
Methyltestosterone (MEST)	0.25 0.50 0.75	0.22 0.42 0.77	0.031 0.057 0.089	88.0 84.0 102.7	13.88 13.51 11.65
Boldenone (BOLD)	0.5 1.0 1.5	0.45 1.09 1.20	0.056 0.142 0.176	90.0 109.2 80.4	12.46 13.08 14.66
19 Nortestosteron (19 NO)	0.25 0.5 0.75	0.19 0.53 0.65	0.016 0.065 0.066	76.2 106.4 86.7	8.57 12.35 10.12
Stanozolol (STZL)	0.25 0.5 0.75	0.24 0.52 0.73	0.028 0.075 0.101	96.3 104.7 97.3	11.84 14.46 13.50
Clostebol (CLBL)	7.5 15 22.5	7.10 14.95 18.65	0.87 2.30 2.96	94.7 99.7 82.9	12.16 15.38 15.89
Zeranol (ZENL)	0.5 1.0 1.5	0.47 0.80 1.36	0.074 0.122 0.121	94.1 80.2 90.7	15.88 15.29 8.90
Taleranol (TANL)	0.5 1.0 1.5	0.42 0.98 1.35	0.057 0.145 0.156	84.5 98.4 90.2	13.65 14.80 11.54
Clenbuterol (CLEN)	0.05 0.1 0.15	0.05 0.08 0.14	0.005 0.011 0.014	94.0 84.2 92.7	11.54 12.82 10.14
Brombuterol (BROM)	0.05 0.1 0.15	0.04 0.10 0.12	0.006 0.012 0.019	82.0 95.0 82.7	14.90 12.56 15.58
Mabuterol (MABT)	0.05 0.1 0.15	0.04 0.08 0.15	0.005 0.012 0.023	84.2 81.0 97.3	12.35 14.46 15.80
Clenpenterol (CLEP)	0.05 0.1 0.15	0.04 0.09 0.15	0.006 0.010 0.022	84.0 86.0 101.3	14.47 11.10 14.22
Isoxuprin (ISOX)	0.25 0.50 0.75	0.26 0.41 0.76	0.041 0.059 0.105	104.4 82.2 101.2	15.84 14.46 13.85
Cimbuterol (CIMB)	0.05 0.1 0.15	0.05 0.08 0.14	0.007 0.008 0.021	92.0 84.0 90.7	15.11 9.25 15.56
Ractopamine (RACT)	0.25 0.50 0.75	0.25 0.44 0.72	0.028 0.067 0.106	100.4 88.2 95.3	11.26 15.11 14.88
Salbutamol (SALB)	0.25 0.50 0.75	0.22 0.47 0.62	0.036 0.067 0.079	89.2 93.4 82.8	15.98 14.36 12.68
Zilpaterol HCl (ZILP)	0.25 0.50 0.75	0.24 0.52 0.71	0.038 0.065 0.095	95.2 104.8 95.2	15.95 12.38 13.29
Terbutaline (TERB)	0.25 0.50 0.75	0.22 0.48 0.76	0.029 0.050 0.120	88.4 95.6 101.5	13.26 10.52 15.78
Amoxicillin (AMOX)	7.5 15 22.5	7.0 13.5 21.4	0.71 2.13 3.79	93.3 90.2 95.4	10.15 15.74 17.70
Ampicillin (AMP)	7.5 15 22.5	6.6 16.1 23.4	2.64 1.96 2.14	88.4 107.3 104.2	17.40 12.24 9.15
Benzylpenicillin (BNPC)	7.5 15 22.5	7.0 13.2 19.4	0.43 1.35 1.82	93.6 88.4 86.2	6.14 10.21 9.46
Lincomycin (LINK)	7.5 15 22.5	7.5 13.4 18.9	0.64 2.20 2.82	100.4 89.5 84.7	8.48 16.45 14.94
Tylosin (TYLS)	7.5 15 22.5	7.6 14.8 19.4	0.93 2.43 2.68	101.3 98.7 86.0	12.25 16.41 13.85
Trimethoprim (TRIP)	7.5 15 22.5	7.4 15.7 22.1	1.10 1.96 3.30	98.7 104.8 98.3	15.48 12.46 14.95
Cephapirin (CEPR)	7.5 15 22.5	6.4 12.8 18.9	0.58 1.08 2.37	85.6 85.2 84.4	9.12 8.46 12.52
Tetracycline (TETC)	7.5 15 22.5	6.9 12.5 23.1	0.39 1.64 1.65	92.2 83.7 102.8	5.65 13.10 7.14
Cloxacillin (CLCN)	7.5 15 22.5	7.2 13.8 19.9	0.66 1.68 2.56	96.0 92.0 88.4	9.15 12.20 12.84
Oxacillin (OXIN)	7.5 15 22.5	7.1 14.5 21.0	0.53 1.90 2.57	94.7 96.7 93.3	7.45 13.12 12.25
Cefalexin (CEFA)	7.5 15 22.5	7.0 14.4 23.8	1.20 1.85 2.73	93.3 96.0 105.8	17.11 12.84 11.46
Ceftiofur (CEFT)	7.5 15 22.5	6.9 13.0 21.7	0.79 1.60 3.57	92.0 86.7 96.4	11.41 12.25 16.41
Enrofloxacin (ENRO)	7.5 15 22.5	6.1 12.5 22.3	1.18 2.19 3.01	81.3 83.3 99.1	19.35 17.48 13.51
Ciprofloxacin (CIPR)	7.5 15 22.5	7.4 14.9 22.8	1.49 2.59 3.77	98.7 99.3 101.3	20.12 17.35 16.48
Oxytetracycline (OXTT)	7.5 15 22.5	6.9 15.4 19.9	1.04 2.56 1.62	92.0 102.7 88.4	15.12 10.14 13.18
Sulfachlorpyridazine (SUPZ)	7.5 15 22.5	7.7 16.3 20.8	1.65 2.48 1.94	102.7 108.7 92.4	21.35 15.22 9.31
Sulfadiazine (SUDI)	7.5 15 22.5	6.2 12.5 18.2	1.31 2.15 1.85	82.7 83.3 80.9	21.13 17.11 10.15
Sulfadimethoxine (SUDM)	7.5 15 22.5	7.4 13.8 22.0	0.99 1.26 3.05	98.7 92.0 97.8	13.41 9.12 13.85
Sulfadimidine (SULD)	7.5 15 22.5	6.6 14.7 21.0	0.89 1.70 2.57	88.0 98.0 93.3	13.46 11.54 12.25
Sulfamethoxazole (SULM)	7.5 15 22.5	8.1 15.9 22.5	1.69 2.33 2.52	108.4 106.0 100.2	20.81 14.66 11.22
Carbofuran (CRL)	7.5 15 22.5	7.1 14.6 19.4	0.59 1.63 1.72	94.7 97.3 86.4	8.35 11.14 8.88
Carbaryl (CRB)	7.5 15 22.5	7.1 13.0 18.9	1.17 2.55 2.33	94.7 86.4 84.2	16.46 19.58 12.35
Parathion (PTN)	7.5 15 22.5	7.4 13.8 22.1	1.27 1.80 2.02	98.7 92.1 98.2	17.12 13.01 9.15
Malathion (MTN)	7.5 15 22.5	6.4 13.2 20.1	0.78 1.22 2.04	85.3 88.6 89.3	12.15 9.21 10.15
Diazinon (DNN)	7.5 15 22.5	7.2 13.7 21.2	0.66 1.16 2.37	96.1 91.4 94.0	9.12 8.46 11.2
Dimethoate (DIM)	7.5 15 22.5	6.4 13.0 18.4	1.23 1.80 1.87	85.3 86.7 81.8	19.23 13.81 10.15
Atrazine (ATRZ)	7.5 15 22.5	7.4 13.7 21.3	1.00 1.39 1.81	98.7 91.3 94.7	13.52 10.15 8.48
Permethrin (PEMT)	7.5 15 22.5	7.0 13.5 20.8	1.49 1.86 2.61	93.3 90.0 92.4	21.35 13.78 12.54
Cypermethrin (CIRM)	7.5 15 22.5	6.9 14.7 18.2	1.06 1.54 2.07	92.0 98.0 80.9	15.31 10.48 11.35
Deltamethrin (DELM)	7.5 15 22.5	7.4 15.8 22.9	1.35 2.13 3.32	98.7 105.3 101.8	18.22 13.48 14.51
Coumaphos (COU)	7.5 15 22.5	8.2 14.6 23.4	1.25 1.98 3.07	109.3 97.3 104.0	15.21 13.58 13.11
Dichlorvos (DIRP)	7.5 15 22.5	6.9 14.6 21.8	1.26 2.53 2.95	92.0 97.3 96.9	18.21 17.35 13.54
Chlorpyrifos (CHRS)	7.5 15 22.5	6.6 13.8 19.5	0.50 1.92 1.58	88.0 92.0 86.7	7.54 13.88 8.11
Fenvalerate (FERT)	7.5 15 22.5	7.1 15.8 22.0	0.65 1.82 1.79	94.7 105.3 97.8	9.11 11.52 8.14
Boscalid (BOS)	7.5 15 22.5	7.1 14.6 18.9	0.65 1.77 2.25	94.7 97.3 84.0	9.15 12.11 11.90
Fenthoate (FETE)	7.5 15 22.5	6.7 16.0 22.1	0.97 2.06 2.24	89.3 106.7 98.2	14.42 12.86 10.14
Fenthion (FEON)	7.5 15 22.5	7.1 13.9 18.5	0.50 1.29 1.89	94.7 92.7 82.2	7.11 9.25 10.14
Monocrotophos (MOCR)	7.5 15 22.5	7.5 14.8 22.0	1.04 1.40 1.78	100.0 98.7 97.8	13.84 9.48 8.11
Malaoxon (MAON)	7.5 15 22.5	6.7 12.9 22.3	1.35 1.69 2.00	89.3 86.0 99.1	20.11 13.11 8.95
Methamidophos (MEDF)	7.5 15 22.5	7.0 12.8 20.9	1.42 2.23 2.69	93.3 85.3 92.9	20.22 17.46 12.88
Metacrifos (MECF)	7.5 15 22.5	6.4 12.5 22.1	0.52 1.17 1.79	85.3 83.3 98.2	8.12 9.36 8.10
Amitraz (AMRZ)	7.5 15 22.5	7.0 14.1 18.3	1.49 1.86 1.80	93.3 94.0 81.3	21.35 13.22 9.88
Omethoate (OMAT)	7.5 15 22.5	6.2 13.1 19.9	0.50 0.99 2.26	82.7 87.3 88.4	8.11 7.52 11.35
Vamidothion (VAON)	7.5 15 22.5	7.4 14.6 22.1	1.23 1.75 2.32	98.7 97.3 98.2	16.60 12.02 10.51
Phosmet (FOST)	7.5 15 22.5	7.4 14.6 18.8	0.90 1.22 1.71	98.7 97.4 83.6	12.20 8.36 9.11
Heptenophos (HEPH)	7.5 15 22.5	6.6 13.2 21.7	0.83 1.21 2.95	84.8 88.2 96.4	12.51 9.15 13.61
Bifenthrin (BFNT)	7.5 15 22.5	6.9 12.8 23.4	0.97 1.17 1.71	92.2 85.3 104.6	14.11 9.12 7.35
Methomyl (MEML)	7.5 15 22.5	7.5 13.6 24.3	1.60 2.07 2.27	100.2 91.0 108.1	21.38 15.22 9.35
Zearalenone (ZEAN)	7.5 15 22.5	7.1 14.0 19.6	0.89 1.43 1.20	94.6 93.4 87.1	12.58 10.22 6.11
Ochratoxin A (OTAA	7.5 15 22.5	6.7 13.6 18.7	1.24 2.00 2.10	89.3 90.6 83.1	18.54 14.65 11.22

### Method validation

#### Linearity

Very good linearity was attained with coefficient of correlation (R^2^) from 0.991 for methylthiouracil and fenvalerate to 0.999 for sulfadimidine, phosmet and ochratoxin A. The range of calibration curve and R^2^ values for all compounds are presented in Table 2.

#### LOD, LOQ, CCα, CCβ

The results for LOD, LOQ, CCα and CCβ are shown in the Table 3. The LODs were from 0.01 µg/L for cimbuterol to 2.71 µg/L for ampicillin, while the LOQs were from 0.05 µg/L for cimbuterol to 7.52 µg/L for oxacillin. The CCα values ranged from 0.05 µg/L for cimbuterol to 12.11 µg/L for cephalexin, while CCβ values ranged from 0.08 µg/L for clenpenterol to 15.16 µg/L for cephalexin.

### Recovery, repeatability and reproducibility

**Fig. 1:**
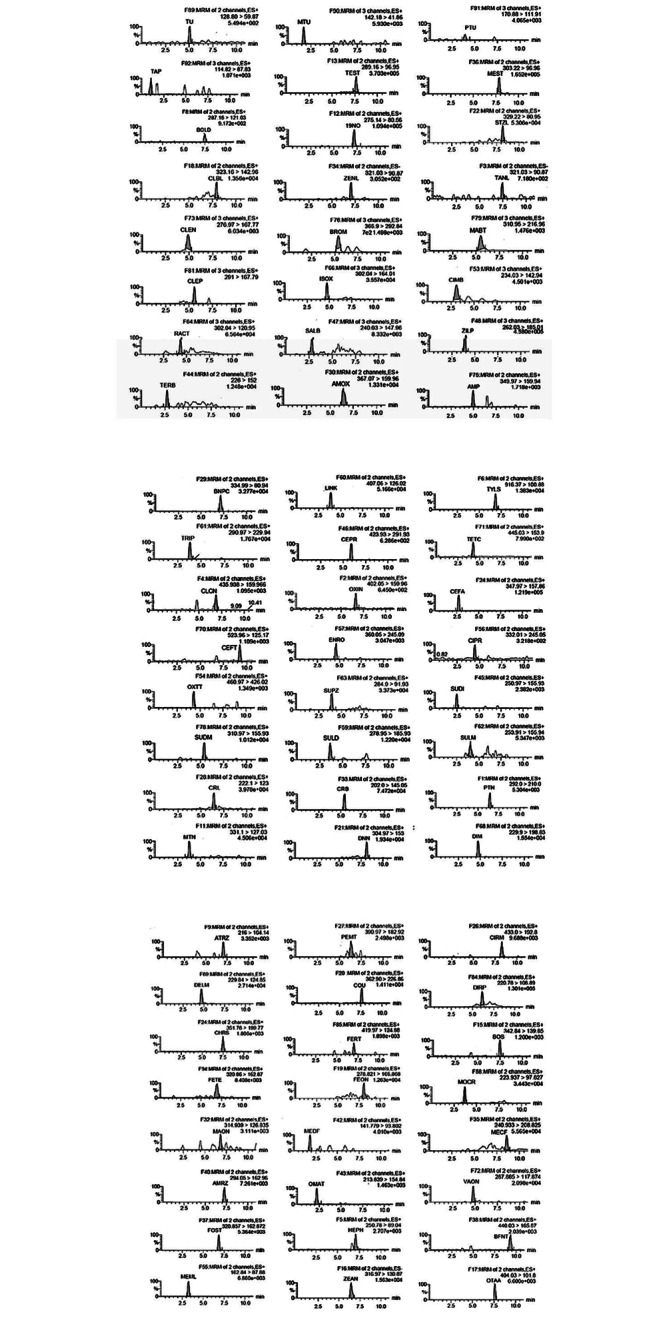
**A** The chromatograms of spiked urine samples - concentration level is level 2 from Table 5 (TU (10 µg/L), MTU (10 µg/L), PTU (10 µg/L), TAP (10 µg/L), TEST (15 µg/L), MEST (0.5 µg/L), BOLD (1.0 µg/L), 19 NO (0.5 µg/L), STZL (0.5 µg/L), CLBL (15 µg/L), ZENL (1.0 µg/L), TANL (1.0 µg/L), CLEN (0.1 µg/L), BROM (0.1 µg/L), MABT (0.1 µg/L), CLEP (0.1 µg/L), ISOX (0.5 µg/L), CIMB (0.1 µg/L), RACT (0.5 µg/L), SALB (0.5 µg/L), ZILP (0.5 µg/L), TERB (0.5 µg/L), AMOX (15 µg/L), AMP (15 µg/L)). **B** The chromatograms of spiked urine samples - concentration level is level 2 from Table 5 (BNPC, LINK, TYLS, TRIP, CEPR, TETC, CLCN, OXIN, CEFA, CEFT, ENRO, CIPR, OXTT, SUPZ, SUDI, SUDM, SULD, SULM, CRL, CRB, PTN, MTN, DNN, DIM − 15 µg/L for all). **C** The chromatograms of spiked urine samples - concentration level is level 2 from Table 5 (ATRZ, PEMT, CIRM, DELM, COU, DIPR, CHR, FERT, BOS, FETE, FEON, MOCR, MAON, MEDF, MECF, AMRZ, OMAT, VAON, FOST, HEPH, BFNT, MEML, ZEAN, OTAA − 15 µg/L for all)

Recovery, repeatability (intraday) and reproducibility (interday) were used for evaluation of the accuracy of the method. The intraday recovery range was from 71.0% for mabuterol spiked at 0.1 µg/l to 117% for cimbuterol at 0.1 µg/L, while the interday recovery range was from 76.2% to 19 nortestosterone spiked at 0.25 µg/L to 109.3% for coumaphos spiked at 7.5 µg/ L. The CV for repeatability ranged from 1.69% for tetracycline to 16.76% for sulfadiazine, while the CV for reproducibility was from 5.65% for tetracycline to 21.38% for methomyl. The results are summarized in Tables 5 and 6. The chromatograms of the analytes in urine samples spiked with standards at concentration level two from Table 5 are shown in Fig. 1 (a, b, c).

### Stability

Stock standards of the anabolic hormones, lactones, beta agonists, thyreostats, mycotoxins and most antibiotics (except amoxicillin, ampicillin, benzylpenicillin, cloxacillin and oxacillin) were stable for 6 months at -20 °C, while pesticide standards were stable for 1 year, and amoxicillin, ampicillin, benzylpenicillin, cloxacillin as well as oxacillin were stable for 3 months. Working standard solutions were stable for 1 month when kept at -20 °C. The standards in the final extract were stable 3 days.

## Discussion

The testing of forbidden substances in media such as urine is an important task of regulatory authorities and laboratories. In the European Union, the use of antimicrobials, thyreostats, anabolic hormones and β-agonist drugs for fattening slaughter animals has been banned since 1981 under Council Directive 81/602/EEC [31]. For protection of consumer health against unwanted residues and in compliance with Directive 96/23, each EU country must monitor these substances in samples of animal origin. One precursor and two product ions were selected for each analyte as recommended elsewhere [32] and most were analyzed in positive ionization mode, except zeranol, taleranol, zearalenone and β-zearalenol-d4 analyzed in the negative ionization mode. The most abundant product ion was used for quantification, while the second product ion was used for confirmation.

Urine is widely used to monitor the illegal use of growth-promoting agents and veterinary drugs with a good number of these substances showing high clearance rates in urine [33–35]. While preparation of urine can generally be easier than for other matrices, sample preparation for analysis of mixed hazards is a challenge to many laboratories thus requiring rigorous extraction and cleanup [10]. The simplest methods for detection of pesticides in urine are direct injection of urine samples or dilute-and-shoot procedures although urinary salts or macromolecules decrease instrument sensitivity, clog the injection syringe or the ESI probe [19, 36]. То avoid such effects solid phase extraction and liquid-liquid extraction procedures are applied for residues of veterinary drugs and contaminants [36–38].

Six extraction protocols were investigated. In the first and second protocols involving LLE, thyreostats were not detected due to poor recovery. The results were comparable with previous studies by Kellman et al. (2009) and Eeckhaut et al. (2009) who concluded that while the LLE is simpler and easier than SPE, matrix (urine) interferences lead to low extraction efficiency hence the need for SPE [39, 40]. Gómez-Pérez et al. (2015), used Florisil cartridges for determination of pesticide and veterinary drug residues, Kaufman et al. (2008), used Oasis HLB cartridge for detection of different classes of veterinary drugs, while Kaklamanos et al. (2009), used Oasis HLB and Amino Supelclean NH_2_ cartridges for analysis of anabolic steroids [12, 38, 41].

Furthermore, Ho et al. (2006), used C8-SCX mixed-mode cartridge for analysis of anabolic steroids, corticosteroids and acidic drugs, while Leon et al. (2012), used Oasis HLB cartridges in a method for 87 analytes in different families of banned or unauthorized substances [42, 43]. In this study SPE extraction involved Oasis HLB and DSC-MCAX cartridges in procedures 3, 4, 5 and 6. Better recoveries can be attained when OASIS HLB cartridges are used along with enzymatic hydrolysis. Kinsella et al. (2009), reported that steroids in urine can be present in free, glucuronic acid and sulphate forms [44]. Also, phenolic β-agonists contain conjugated esters, especially in the form of glucuronides and sulphates. Enzymatic hydrolysis (with β-glucuronidase aryl sulfatase) deconjugate steroid glucuronides and sulfates and this can improve recovery [44, 45]. Overall, the validation parameters demonstrate suitability of the method to analyse a mixture of residues/contaminants such as veterinary drugs, pesticides and mycotoxins in bovine urine, and in agreement with criteria described in 2021/808/EC [25] and is applicable to field /real sample analysis.

### Real sample analysis

**Fig. 2:**
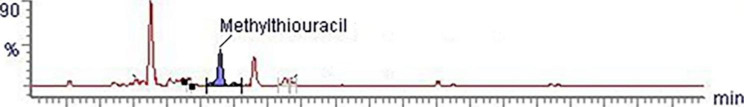
Chromatogram – Methylthiouracil in bovine urine

**Fig. 3:**
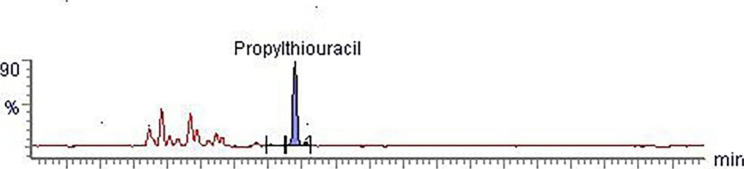
Propylthiouracil in bovine urine

A total of 83 bovine urine samples were collected from local farms and tested using the developed and validated method during 2021–2022. In five samples of urine, methyl- and propylthiouracil were found. No other veterinary drug residues and contaminants were found. Methylthiouracil (veterinary drug) residue amount was found between 1.08 and 1.31 µg/L in 5 samples and propylthiouracil (veterinary drug) amount was found to be 1.67–2.63 µg/L in 4 samples. In Fig. 2 is given chromatogram of bovine urine with methylthiouracil, while in Fig. 3 is given chromatogram of bovine urine with propylthiouracil. The presence of thiouracil and its derivatives in urine samples is most likely due to feeding animals diet containing cruciferous plants. The proposed method, consisting of enzymatic hydrolysis using β-glucuronidase and cleanup solid phase extraction with OASIS SPE cartridges, allowed analysis at low level concentrations without any matrix interference for all samples, indicating that the method was very effective for regulatory monitoring of bovine urine for 72 residues of veterinary drugs residues, pesticides and mycotoxins.

## Conclusion

A new isotopic LC-MS/MS method has been developed, validated and applied for identification and quantification of 72 residues of veterinary drugs and pesticides and other contaminants such as mycotoxins in bovine urine. The most appropriated sample preparation procedures involved sodium acetate buffer, enzymatic hydrolysis using β-glucuronidase and cleanup solid phase extraction with OASIS SPE cartridges. The parameters were satisfactorily validated fulfilling requirements under Regulation 2021/808/EC. Consequently, the method could be used in routine analysis of bovine urine samples for simultaneous detection of veterinary drug and pesticide residues as well as contaminants such as mycotoxins.

## Data Availability

All data and materials analyzed during the current study are included in the manuscript.

## Abbreviations

CCα: Decision limit
CCβ: Detection capability
CV_r_: Coefficient of variation for repeatability
CV_R_: Coefficient of variation for reproducibility
EC: European Commission
EURL: European Union Reference Laboratory
LC-MS/MS: Liquid chromatography tandem mass spectrometry
LLE: Liquid-liquid extraction
LOD: Limit of detection
LOQ: Limit of quantification
MMPRs: Minimum method performance requirements
MRM: Multiple reaction monitoring
MRLs: Maximum residue limit
PBS: Phosphate buffer
R^2^: Coefficient of correlation
S/N: Signal to noise
SPE: Solid phase extraction
USA: United States of America
